# A Novel Photoswitchable Azobenzene-Containing Local Anesthetic Ethercaine with Light-Controlled Biological Activity In Vivo

**DOI:** 10.3390/ijms23105352

**Published:** 2022-05-11

**Authors:** Alexey Noev, Nikita Kuznetsov, Georgiy Korenev, Natalia Morozova, Yuriy Vasil’ev, Nikita Suvorov, Ekaterina Diachkova, Maksim Usachev, Andrei Pankratov, Mikhail Grin

**Affiliations:** 1 Department of Chemistry and Technology of Biologically Active Compounds, Medicinal and Organic Chemistry, Institute of Fine Chemical Technologies, MIREA-Russian Technological University, 86 Vernadsky Avenue, 119571 Moscow, Russia; drtimmy85@yandex.ru (N.K.); gcorenev@mail.ru (G.K.); suvorov.nv@gmail.com (N.S.); maximus021989@mail.ru (M.U.); michael_grin@mail.ru (M.G.); 2 P. Hertsen Moscow Oncology Research Institute—Branch of the National Medical Research Radiological Centre of the Ministry of Health of the Russian Federation, 2nd Botkinsky pr. 3, 125284 Moscow, Russia; n.b.morozova@yandex.ru (N.M.); andreimnioi@yandex.ru (A.P.); 3 Department of Topographic Anatomy and Operative Surgery, Sklifosovskii Institute of Clinical Medicine, I.M. Sechenov First Moscow State Medical University (Sechenov University), Trubetskaya St. bldg. 8\2, 119435 Moscow, Russia; vasilev_yu_l@staff.sechenov.ru; 4 Department of Prosthetic Dentistry, Dental Faculty, Kazan State Medical University of the Ministry of Health of Russia, Str. Butlerova 49, 420012 Kazan, Russia; 5 Department of Oral Surgery Borovskiy Institute of Dentistry, I.M. Sechenov First Moscow State Medical University (Sechenov University), Trubetskaya St. bldg. 8\2, 119435 Moscow, Russia

**Keywords:** ethercaine, fotocaine, photopharmacology, photoswitchable local anesthetic, azobenzene, micelles, corneal anesthesia

## Abstract

Pain is a common symptom that impairs the quality of life for people around the world. Local anesthetics widely used for pain relief have a number of side effects, which makes the development of both new drugs and new ways to control their activity particularly important. Photopharmacology makes it possible to reduce the side effects of an anesthetic and control its biological activity in the body. The purpose of this work was to create a new light-controlled local anesthetic and study its biological activity in animals. A compound with a simple scheme of synthesis was chosen to shift the UV-Vis absorption band towards the visible range of the spectrum and was synthesized for the first time. Some computer calculations were performed to make sure that the aforementioned changes would not lead to loss of biological activity. The micellar form of the new compound was prepared, and in vivo biological studies were carried out in rabbits. The existence of a local anesthetic effect, which disappeared almost completely on irradiation with light (λ = 395 nm), was shown using the surface anesthesia model. Moreover, the possibility of multiple reversible changes in the biological activity of ethercaine under the action of light was demonstrated. The latter compound manifests no local irritating effect, either. The data obtained indicate the prospects for the development of new compounds based on azobenzene for light-controlled local anesthesia.

## 1. Introduction

**Figure 1:**
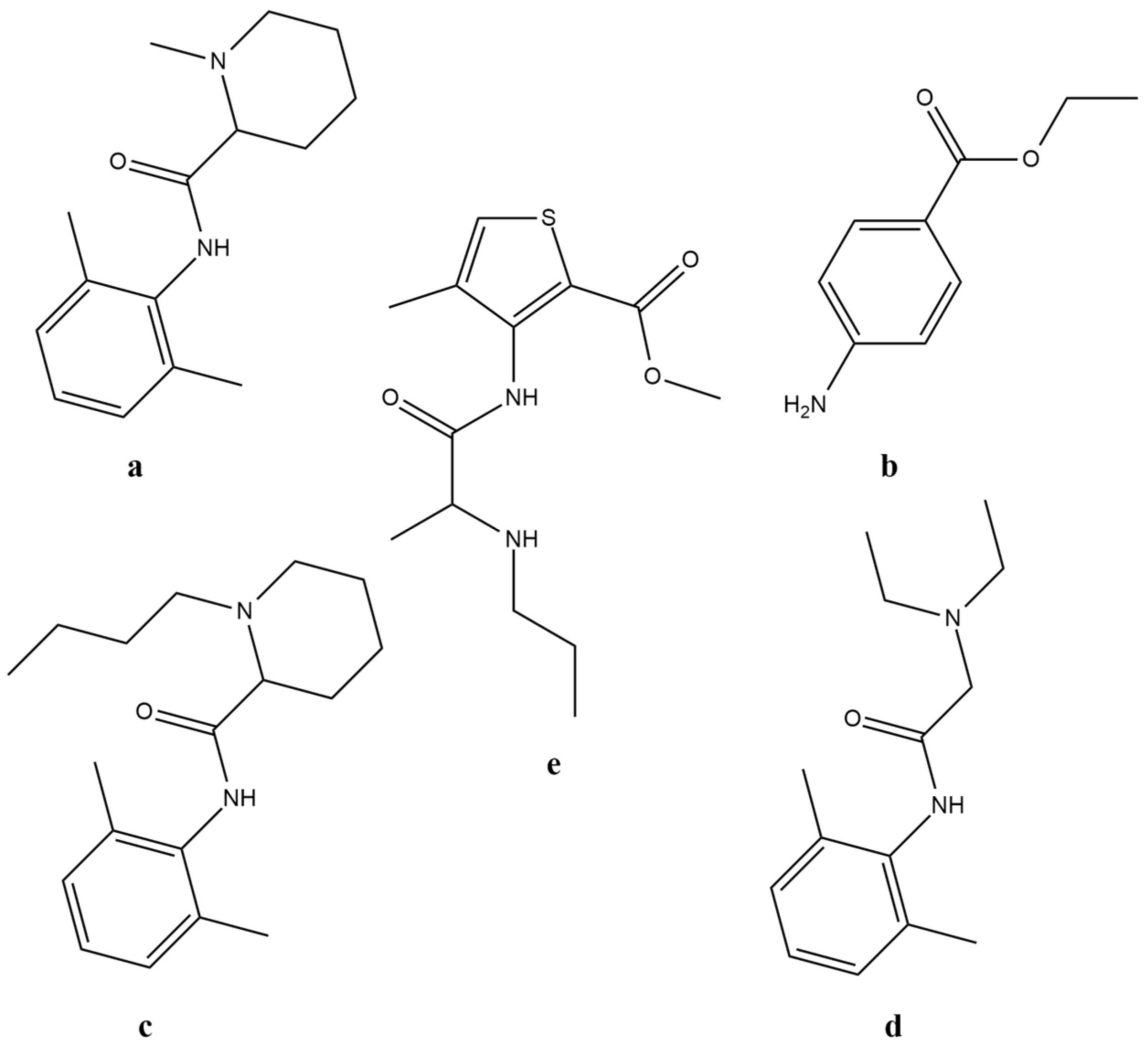
Chemical structures of the most common local anesthetics: mepivacaine (**a**), benzocaine (**b**), bupivacaine (**c**), lidocaine (**d**) and articaine (**e**).

Pain is one of the most common pathological conditions that can significantly impair the quality of a patient’s life. The use of local anesthetics (LA) is a traditional method of pain relief, along with opioids and non-steroidal anti-inflammatory drugs. At the same time, according to statistics, about 46% of patients have at least one risk factor if they use local anesthetics, and the incidence of side effects is about 4.5% of all use cases [1,2]. Over the past century, many LA molecules have been invented, including widely used ones such as articaine, lidocaine, procaine, benzocaine, mepivacaine, and bupivacaine, etc. (Figure 1) [3].

Almost all clinically approved LA are non-selective blockers of voltage-gated sodium channels. They act by blocking the transmission of the action potential in peripheral neurons. Despite the advances in the development of new LA, their clinical application still has a number of drawbacks. The effect of anesthetics outside the area of therapeutic interest or the local side effects can lead to a wide range of various complications, including systemic toxicity, cardiotoxicity, local allergic reactions, anaphylaxis, paresthesia and a local irritative effect [4,5,6,7,8,9]. All of this dictates the need for spatiotemporal control of local anesthesia that might allow doctors to control the effect of a drug on the patient’s body.

Several strategies, one of which involves the use of the controlled drug delivery system (DDS), including nanomedical constructs such as liposomes, nanoparticles and microgel systems loaded with anesthetics, have been developed to overcome the LA drawbacks outlined above [10,11,12]. In addition to ultrasonic, magnetic or electrical exposure, light is one of the most promising ways to control the effect of drugs [13,14]. Light radiation is a non-invasive tool for monitoring the local anesthetic activity inside a body. Photosensitive nanocarriers for pain relief, including liposomes loaded with tetrodotoxin (TTX), liposomes containing a photosensitizer and gold nanorods, and a photosensitive microgel system with ropivacaine and a light-controlled microneedle system loaded with lidocaine, were reported in the literature [15,16,17]. However, another approach to light-controlled anesthesia involves the use of a prodrug strategy in which an anesthetic is modified with light-degradable chemical moieties and linkers. Examples of such compounds include light-activated analogs of propofol, tetracaine, and morphine [18,19,20]. In 2017, J. Font et al. reported the creation of a prodrug based on the mGlu5 receptor inhibitor that showed light-dependent activity in models of chronic and acute pain in mice [21]. Recently, the same scientific team created a photodegradable prodrug based on morphine, which made it possible to implement light-activated anesthesia without side effects inherent in opioids [22]. However, despite the significant advances in this field, light irradiation of anesthetics loaded into DDS and the use of photolabile prodrugs results in an irreversible drug release and a pharmacological effect.

In addition to the improvement of delivery methods for well-known drugs, light makes it possible to develop new pain control strategies, including optogenetics and photopharmacology [23,24,25]. Currently, optogenetics is successfully used to study various ways of transmitting pain signals [23,26,27]. This method is based on the directed expression of photosensitive proteins (rhodopsins, bacteriorhodopsins, etc.) in certain types of body cells, depending on the selected promoter [28]. In particular, examples of using this approach to monitor the activity of Na_v_1.8- expressing nociceptive afferents, which makes it possible to control pain in mice, were reported [27,29]. However, the need for the targeted impact on the genome is the main limitation for the application of this method in clinical practice.

Photopharmacology is among the most promising methods to control the activity of drugs inside a body by means of light [30]. The essence of this method involves creating agents capable of reversible transition between two forms, one of which has a biological activity, whereas the other one has none. To date, several photoswitchable compounds are known that can act as blockers of nerve impulse transmission, including photoswitchable modulators of metabotropic glutamate receptors 4 (mGlu4) and 5 (mGlu5), azo-propofols, azo-derivatives of fentanyl, a photoswitchable inhibitor of glycine transporter 2, and azo derivatives of capsaicin [31,32,33,34,35,36,37]. The most promising photoswitchable blockers of voltage-gated sodium channels (Na_v_) to date are QAQ and fotocaine [38,39].

However, the ability of the QAQ molecule to penetrate through the cell membrane is limited, which significantly limits its applications. In 2014, D.Trauner’s group reported the creation of fotocaine, an azologized derivative of the local anesthetic fomocaine [39]. Fotocaine was found to be a promising Na_v_ channel blocker; however, no continuation of studies on the local anesthetic activity of fotocaine in vivo is available in the literature.

In this paper, we report on the synthesis of a new fotocaine derivative and a study of its local anesthetic activity using a model of surface anesthesia in rabbits.

## 2. Results and Discussion

### 2.1. Design

**Figure 2:**
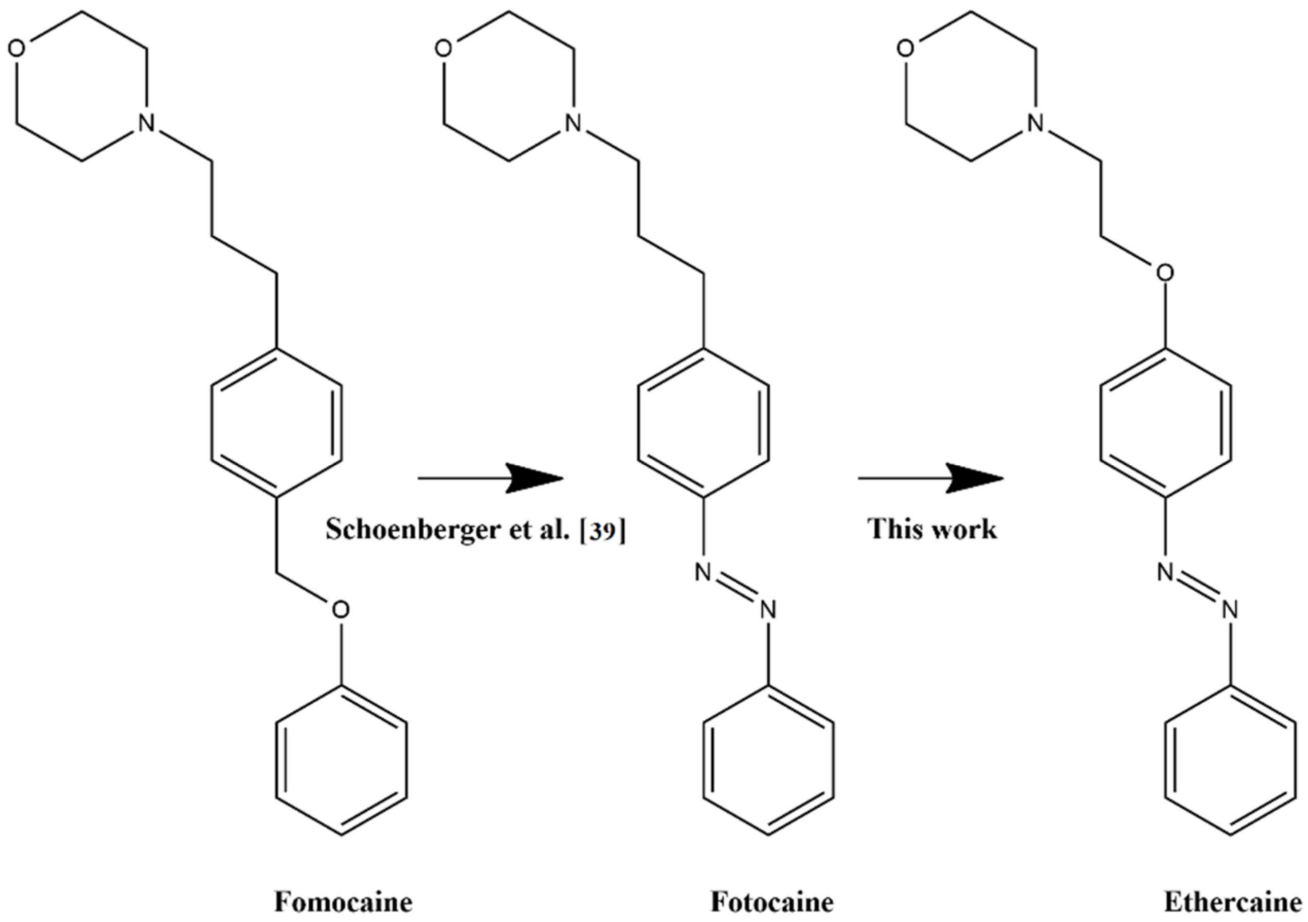
Optimization of the photoswitchable anesthetics’ chemical structures.

We chose a fotocaine analogue, an azobenzene ether, as a key compound in this work. This compound that we named ethercaine has the 4-(2-(*N*-morpholino)-ethoxy)-azobenzene structure (Figure 2).

Replacement of the methylene group closest to the aromatic ring with an ether group causes a bathochromic shift of the characteristic absorption band in the electronic spectrum of ethercaine. Thus, one can expect an improvement in spectral characteristics that would allow switching between the isomers at the border between UV and visible range, which can reduce the adverse effects of UV light on the body and increase the depth of light penetration into tissues.

**Figure 3:**
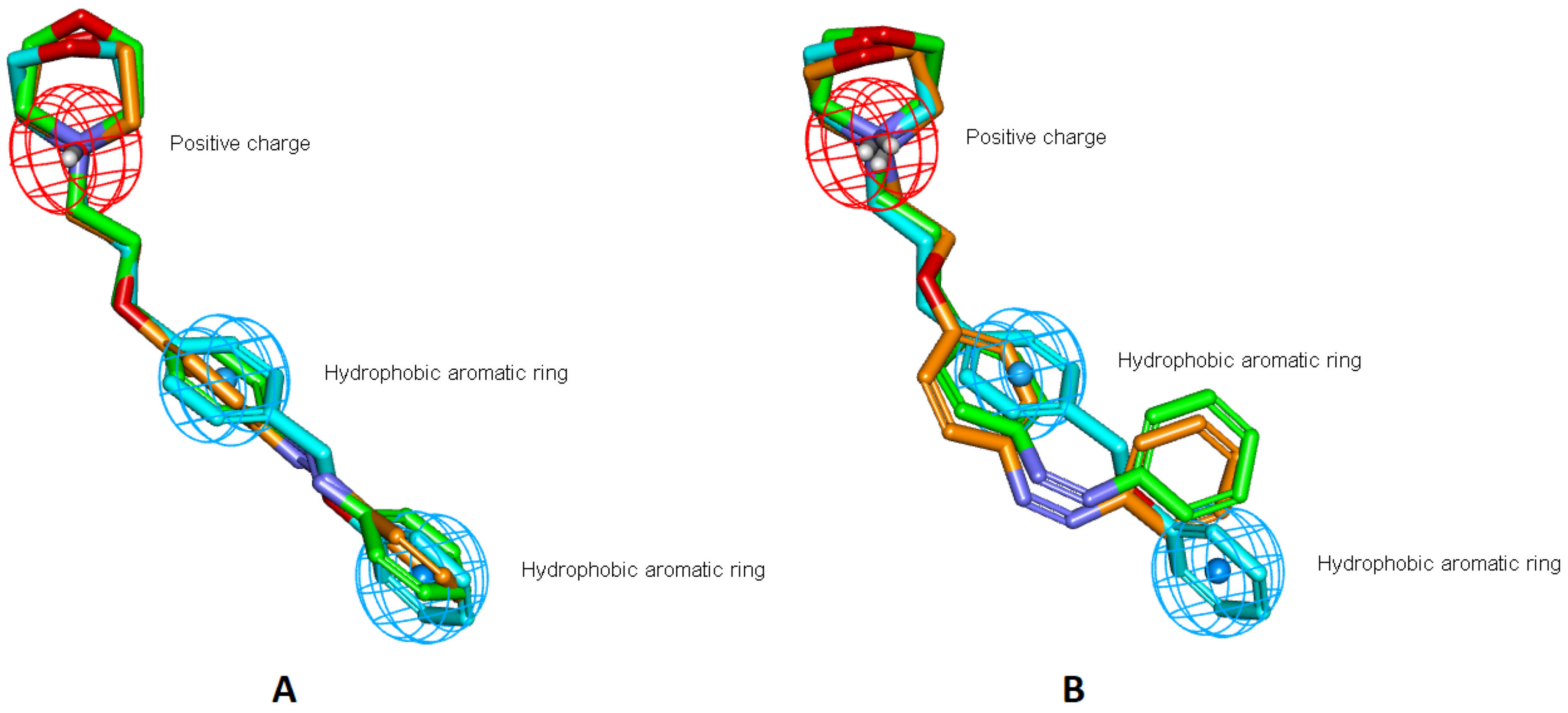
Comparison of predicted 3D structures of fotocaine and ethercaine in *trans* (**A**) and *cis* (**B**) forms. Fotocaine, ethercaine, and fomocaine are shown in green, orange, and cyan, respectively.

A comparison of the calculated 3D structures of the *E*-isomers of ethercaine and fotocaine with fomocaine shows that all the main pharmacophore points retain their spatial positions (Figure 3). Calculated 3D structures of the *Z*-isomers of ethercaine and fotocaine are similar to each other and differ from fomocaine.

Analysis of the spatial structures of the compounds allowed us to assume that ethercaine would have photoswitching properties and retain activity only in the *trans* form, similarly to fotocaine.

**Figure 4:**
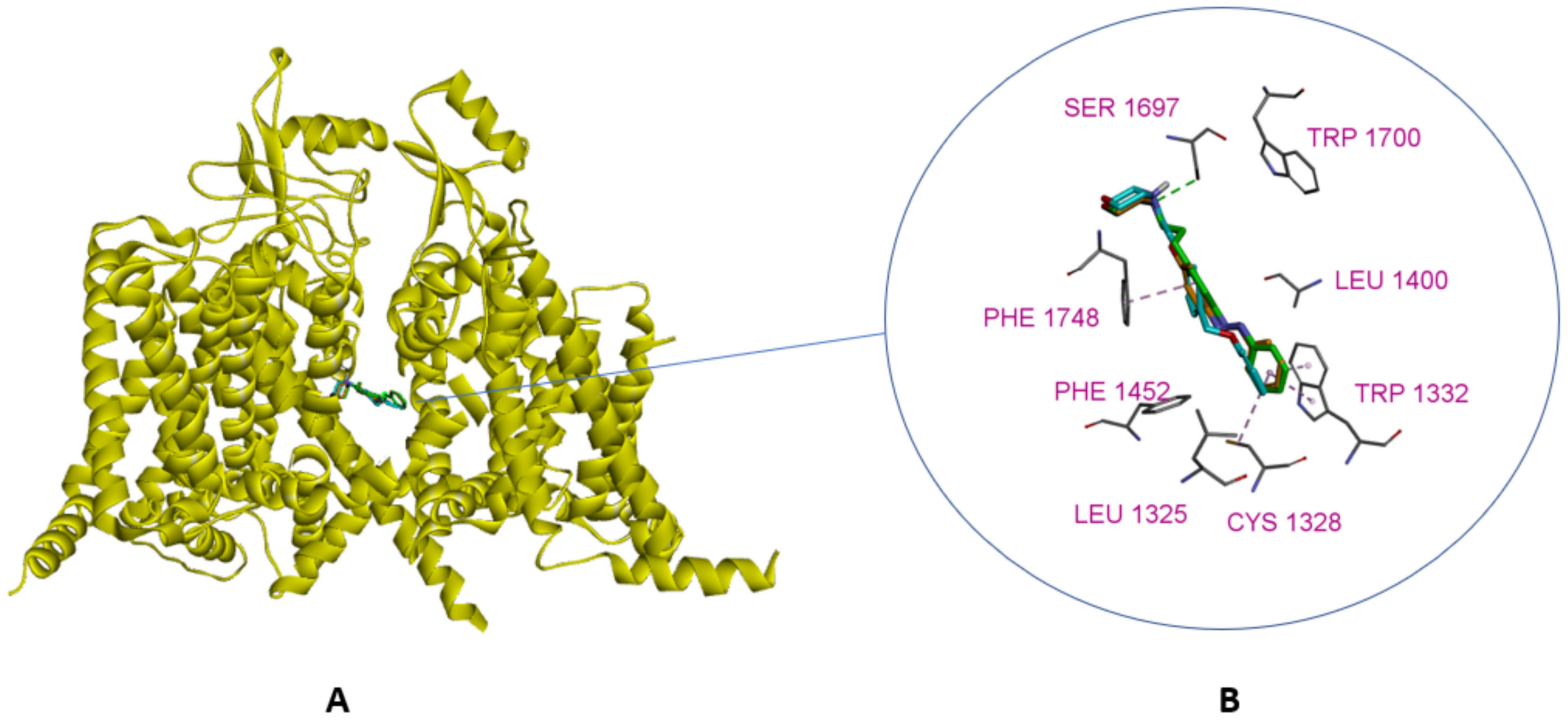
(**A**) Structure of the Na_v_1.7 channel (PDB ID: 6J8H) with docked structures of fotocaine, ethercaine, and fomocaine (shown in green, orange, and cyan, respectively). (**B**) Ligand interactions with the binding site of the Na_v_1.7 channel.

Molecular docking calculations were performed to evaluate the possibility of ethercaine binding to the voltage-gated sodium channels. Local anesthetics bind in the inner part of the pore region of Na_v_ channels, which is known to have a high degree of sequence identity among all known Na_v_ isoforms. Na_v_1.7 isoform is a genetically validated target for pain treatment [40], so the structure of this isoform (PDB ID: 6J8H, [41]) was used to further calculations (Figure 4).

Obtained results demonstrate similar binding poses for fomocaine, fotocaine, and ethercaine without significant differences. Exploring ligand interactions reveal the interaction with Phe1748 (numbering for the canonical Nav1.7 isoform sequence, in some protein isoforms, this residue is numbered Phe1737). This residue is conserved among Na_v_ isoforms and is believed to be very important for local anesthetic binding [42,43]. The results obtained are consistent with published data on the mode of anesthetic drugs binding to Na_v_ channels [43,44,45].

### 2.2. Chemistry

**Scheme 1:**
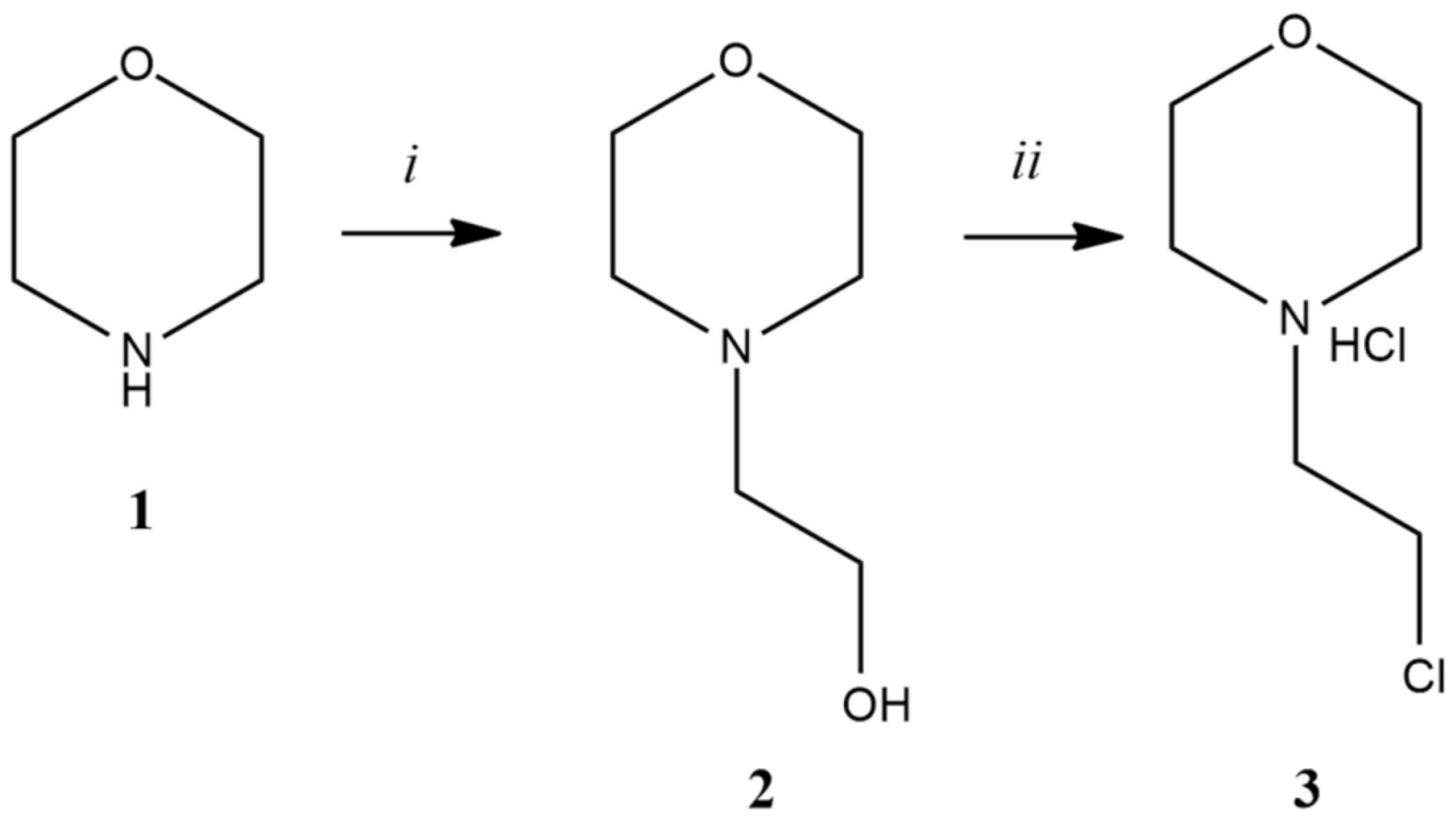
Conditions and reagents: (i) 2-bromoethanol, K_2_CO_3_, CH_3_CN, reflux, 3 h, yield 63%; (ii) SOCl_2_, CHCl_3_, reflux, 4 h, yield 83%.

The original scheme of fotocaine synthesis involves the nitration stage, which may give regioisomers as side products, and employs an expensive palladium catalyst. The scheme of ethercaine synthesis that we suggested involves the synthesis of *N*-(2-chloroethyl)-morpholine (**3**) and 4-hydroxyazobenzene (**5**) followed by *O*-alkylation. The required *N*-(2-chloroethyl)-morpholine (**3**) was obtained from morpholine (**1**) via intermediate *N*-(2-hydroxyethyl)-morpholine (**2**) (Scheme 1).

**Scheme 2:**
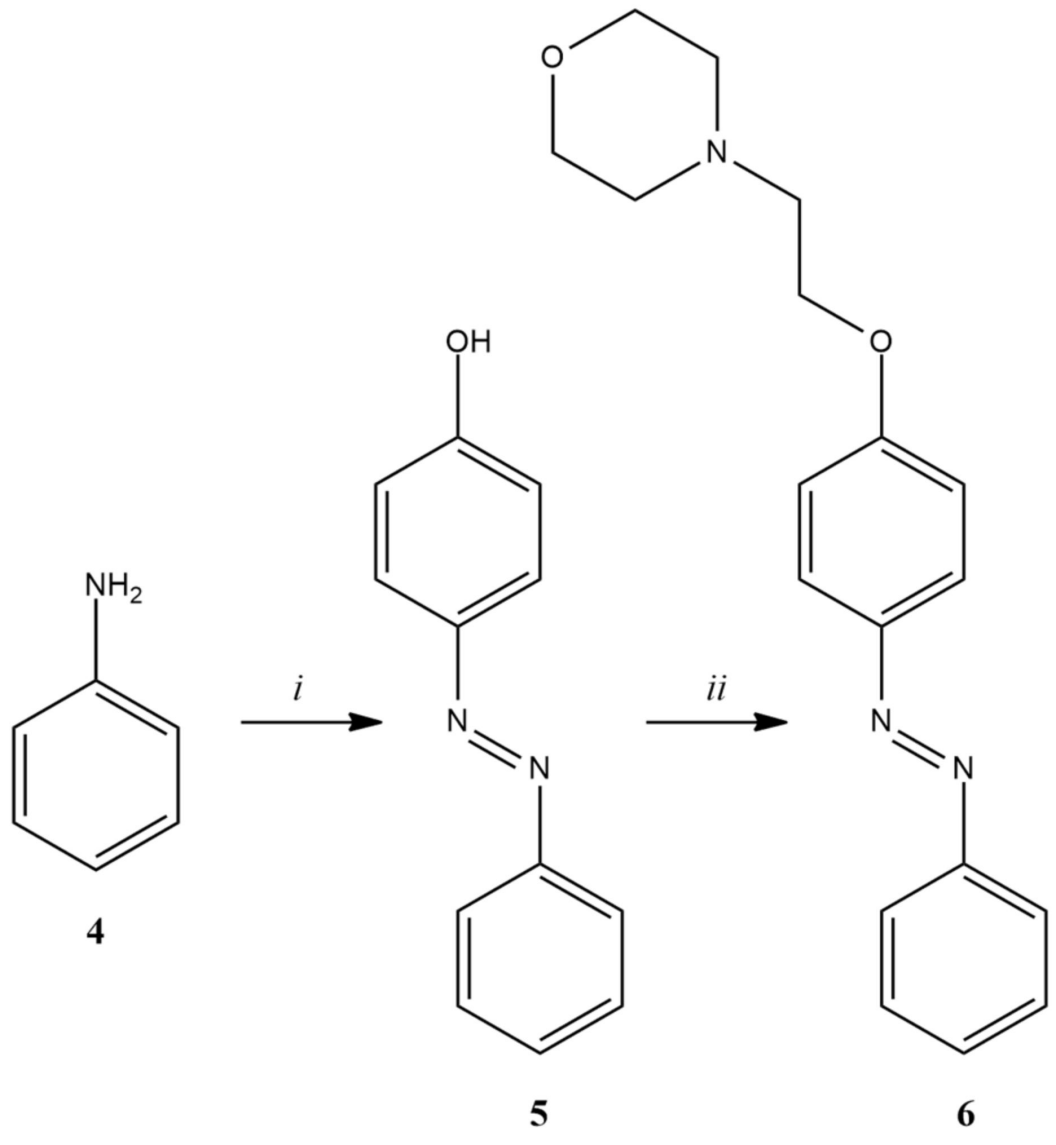
Conditions and reagents: (i) NaNO_2_, HCl/H_2_O, 30 min, then phenol, KOH/H_2_O, 1 h, yield 71%; (ii) (3), K_2_CO_3_, KI, CH_3_CN, reflux, 8 h, yield 85%.

In turn, compound **5** was obtained from aniline **4** by diazotization and azo coupling with phenol. The synthesis of the target compound **6** is demonstrated in Scheme 2.

Ethercaine (**6**) manifests a bathochromic shift of the π-π* absorption band maximum (Δλ ≈ 20 nm, measured in DMSO (Supplementary Materials, Figure S21) compared to the published fotocaine UV/Vis spectrum in the corresponding solvent). Ethercaine (**6**) turned out to be almost insoluble in water and poorly soluble in water/DMSO systems that are suitable for biological studies. It was dissolved in 4% micellar aqueous solution Kolliphor ^®^ ELP, which is a versatile solubilizer for hydrophobic compounds in biological assays. The maximum permissible concentration of the compound such that the solution is stable at room temperature was found to be 0.6%. The micelles thus obtained were characterized by the dynamic light scattering method. The hydrodynamic size derived from measurements by volume showed no statistically significant difference for the micellar solution of *E-* and *Z*-forms of ethercaine. The size of micelles was 16.54 ± 6.11 and 10.45 ± 4.07 nm for *E-* and *Z-* form solutions (the polydispersity indexes were 0.254 and 0.236, respectively).

### 2.3. Photoswitching Properties

**Figure 5:**
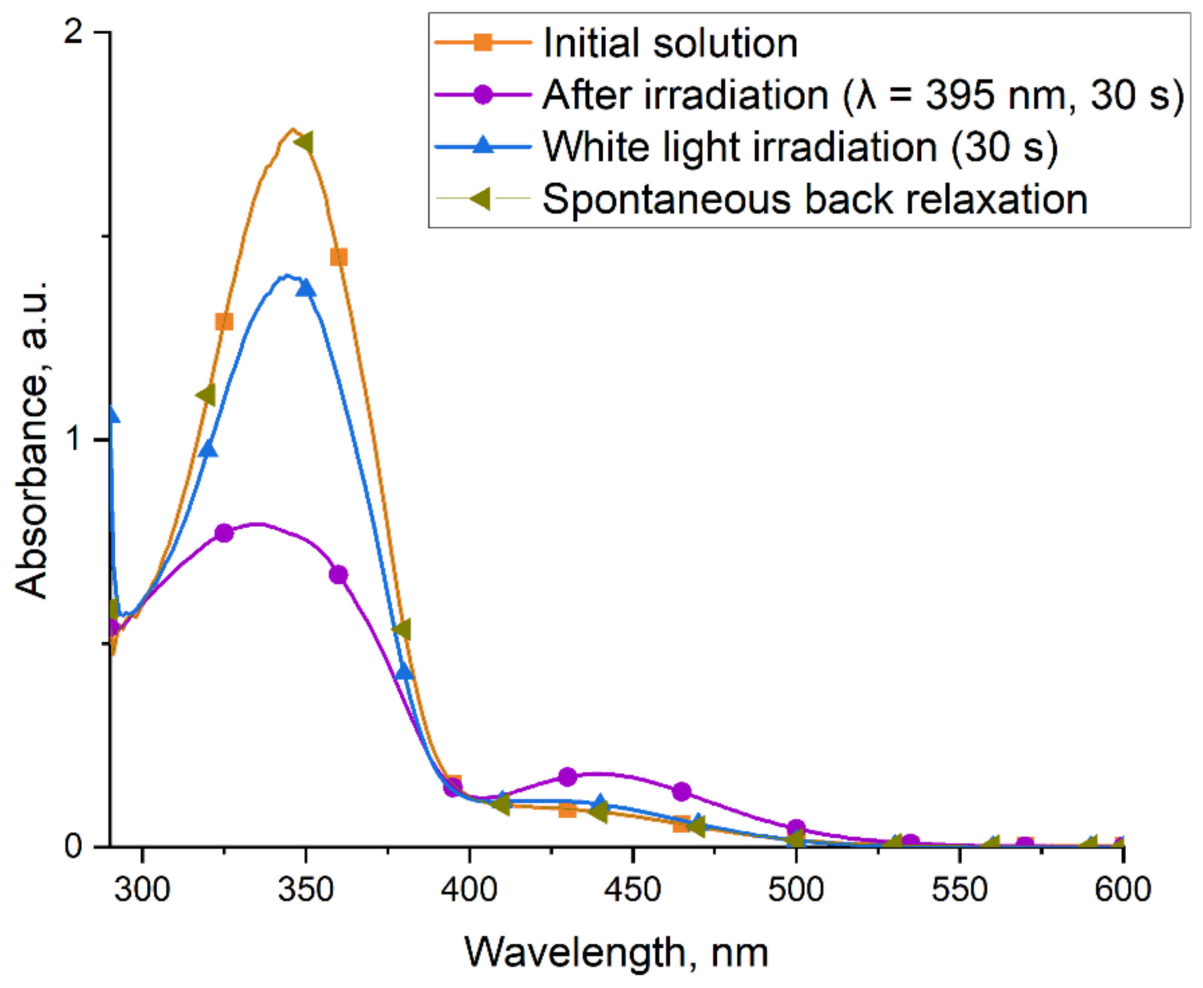
UV/Vis absorption spectra of ethercaine (**6**) before and after irradiation of its solution.

The photoswitching properties of the micellar solution of compound **6** were studied using UV/Vis spectrophotometry. Despite the successful use of UV light in a number of medical procedures [46], exposure to UV-A and UV-B light is still potentially harmful to humans. In order to diminish the possible negative impact of UV radiation on the body in biological studies, a light source with a wavelength of λ = 395 nm, which is at the edge of the visible range, was used. When the cell with a solution is irradiated, a transition to another photostationary state with the predominance of the *Z*-isomer in the solution occurs, as it follows from the changes in the electronic absorption spectrum characteristic of this class of compounds (Figure 5) [39,47,48]. The solution was irradiated stepwise with increasing exposure time, and it was determined that the time sufficient for the equilibrium with the predominance of Z-form is 30 s.

A short-term study of the stability of a solution of the *Z*-form of ethercaine in the dark at room temperature for 5 min did not show any significant changes in the absorption spectrum, which indicates that the *Z*-form of the compound is sufficiently stable. The half-life of the *Z*-form of ethercaine in micellar solution was ca. 65 min (determined by the absorbance at 430 nm on UV-Vis spectra). Irradiation of the solution with regular white light (λ = 420–700 nm) for 30 s led to the recovery of the compound to the photostationary state with the predominance of the more stable *E*-form. Subsequent irradiation with white light did not lead to a change in the absorption spectrum. After several hours, the equilibrium reached the same state as it was at the beginning of the experiment via the back relaxation of the remaining amount of the *Z*-isomer. Thus, irradiation with light at the indicated wavelengths allows compound **6** to be switched reversibly between the *E*- and *Z*-forms.

### 2.4. Biology

In vivo studies are mainly used to estimate the local anesthetic properties of new compounds, since in vitro studies practically do not allow a complex model of pain signal transmission to be simulated. To study the local surface anesthetic effect, the Regnier method was chosen, which involves determining the sensitivity of the cornea of the rabbit’s eye under tactile impact [49].

The addition of 0.6% micellar solution of ethercaine (**6**) to the conjunctival sac of a rabbit led to a pronounced local anesthetic effect lasting 32 ± 5 min. When conducting a similar experiment with light irradiation at a wavelength of λ = 395 nm, nearly no local anesthetic effect was observed. The difference in the Regnier index values for 0.6% ethercaine solution in the presence and in the absence of irradiation reaches one order of magnitude, which indicates that the biological response depends on the presence or absence of irradiation (Table 1).

As expected, the local anesthetic activity of 2% lidocaine solution selected as a control anesthetic manifests no statistically significant differences with and without irradiation. A 4% micellar solution of Kolliphor ^®^ ELP used as a negative control had a minimum Regnier index, which indicated that local anesthesia was completely absent.

With a micellar solution of compound **6,** there were no signs of redness or enlargement of the conjunctiva, sclera, or eyelids, which indicated the absence of a local irritating effect.

**Figure 6:**
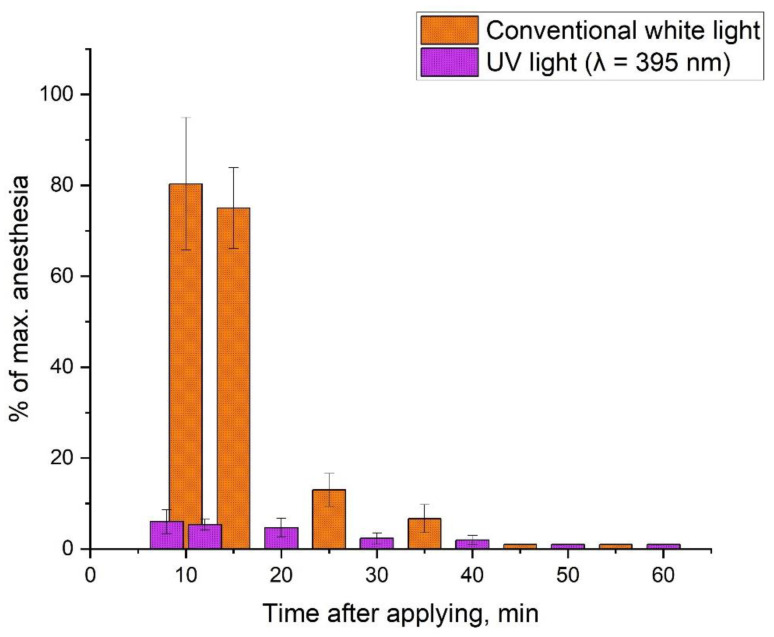
Multiple reversible photoswitching of ethercaine (6) between biologically active and inactive forms in vivo. The orange color shows the values obtained with conventional white light (λ = 420–700 nm), purple—upon irradiation with light at λ = 395 nm. Error bars represent the standard deviations (*n* = 8).

The next stage was to obtain experimental proof of the possibility of multiple reversible photoswitching of ethercaine between the *E*- and *Z*- forms in vivo. To do so, before starting the experiment, the solution was irradiated with light at a wavelength of λ = 395 nm, after which it was instilled in the conjunctival sac of the rabbit’s eye. The first measurement corresponding to 8 min after addition confirms the transition of ethercaine (**6**) to an inactive form under light impact (λ = 395 nm) (Figure 6). Before each subsequent measurement, the eye was irradiated for 30 s by alternating conventional white light (λ = 420–700 nm) and UV light with a wavelength λ = 395 nm.

Based on the data in Figure 6, one can see that ethercaine (6) can reversibly switch between biologically active and inactive forms under the action of light with the corresponding wavelengths. No significant loss of depth and duration of anesthesia was observed for the active form of ethercaine after multiple reversible photoswitching compared to results obtained for the active form of ethercaine in the dark.

## 3. Materials and Methods

### 3.1. Materials

All the chemicals were obtained from commercial sources (Merck KGaA, Darmstadt, Germany; Acros Organics—part of Thermo Fischer Scientific, Waltham, MA, USA) unless specified otherwise and were used without further purification. For micellar solutions preparation Kolliphor ^®^ ELP was used (BASF, Ludwigshafen, Germany). All deuterated solvents were purchased from Cambridge Isotope Laboratories, Inc. (Tewksbury, MA, USA). Organic solvents were purchased from the CHIMMED company (Moscow, Russia), distilled, and dried using standard procedures. Silica gel 60 (Merck KGaA, Darmstadt, Germany) was used for column chromatography. Analytical TLC was performed on aluminum plates with Kieselgel 60 F245 silica gel (Merck KGaA, Darmstadt, Germany).

Electronic absorption spectra were obtained using a Shimadzu UV1800 UV/VIS spectrophotometer (Shimadzu Corporation, Kyoto, Japan) in a 10 mm thick quartz cell. NMR spectra were obtained on a Bruker DPX300 and Bruker Avance 600 spectrometers (Bruker Corporation, Billerica, MA, USA) using CDCl_3_, DMSO-d6 and D_2_O as solvents. Tetramethylsilane or residual solvents were used as the reference standard for spectra calibration. Coupling constants, when given, are reported in hertz (Hz). The hydrodynamic size of micelles was measured using a Zetasizer Nano ZS (Malvern Panalytical, Malvern, United Kingdom).

Analytical HPLC was performed on a Vanquish liquid chromatographic system coupled to a Q-Exactive HF-X high-resolution hybrid mass spectrometer (Thermo Fischer Scientific, Waltham, MA, USA) using a «Pyramid» C18 column (75 mm × 2 mm, 1.8 μm) (Macherey-Nagel, Düren, Germany). Detailed elution and detection conditions are provided in the Supplementary Materials (Table S1). Parameters of the mass spectrometer ionization source are presented in Table S2, and operating parameters of the mass spectrometer modes are presented in Table S3.

Non-anesthetized mature male rabbits of the Soviet chinchilla breed weighing 2–3 kg obtained from the KrolInfo farm (Orekhovo-Zuyevo, Russia) were used in this study. Animals were kept under standard conditions (humidity 50–60%, temperature 19–22 °C). A 12-h lighting cycle was maintained in the animal premises, where each animal was kept in a separate cage. Animals were given ad libitum access to standard extruded feed stuff for rabbits “CHARA” (CJSC “Assortiment-Agro”) and clean drinking water. Water treatment was performed using a “7 TECHNOCOM” block modular system (LLC “7 TECH”). For in vivo surface anesthesia measurements, an in-house anesthesiometer was made. This device had a similar appearance to the Cochet-Bonnet anesthesiometer and had a fixed nylon filament with constant parameters (1.0 cm length, 0.125 mm diameter) at the end.

Statistical analysis was performed and plotted using Origin 9.7 (OriginLab Corporation, Northampton, MA, USA) software. All data are expressed as the mean ± standard deviation.

### 3.2. Design

#### 3.2.1. Ligands 3D-Structures Alignment

The 3D geometry of compounds was optimized using an MM2 force field in Chem3D software (Perkin Elmer Informatics, Inc., Waltham, MA, USA). Alignment and graphical presentations were made using a Discovery Studio Visualizer (version 21.1.0.20298, Dassault Systems Biovia Corp., San Diego, CA, USA).

#### 3.2.2. Molecular Docking Calculations

The crystal structure of the Na_v_1.7 channel was obtained from the Protein Data Bank (PDB ID: 6J8H). The morpholine nitrogen atom was manually protonated and the geometries were minimized using an MM2 force field in Chem3D software (Perkin Elmer Informatics, Inc., Waltham, MA, USA). All of the torsional bonds of the ligands were free to rotate, while protein was held rigid. The Gasteiger partial charges were calculated, atom types were assigned, and non-polar hydrogen atoms were merged using the AutoDockTools 1.5.7 (The Scripps Research Institute, La Jolla, CA, USA). A grid box of dimensions 40 × 40 × 40 with a spacing of 1 Å was created and centered on the mass center of the ligand. Energy grid maps for all possible ligand atom types were generated by using Autogrid 4 before performing docking. The Autodock 4.2.6 (The Scripps Research Institute, La Jolla, CA, USA) was used for docking calculations using the Lamarckian genetic algorithm (LGA) [50]. Ligand interactions were determined, and graphical representations were made using the Discovery Studio Visualizer (version 21.1.0.20298, Dassault Systems Biovia Corp., San Diego, CA, USA).

### 3.3. Synthesis

#### 3.3.1. Synthesis of N-(2-Ethanol)-Morpholine (**2**)

Morpholine (1) (10.00 g, 114.81 mmol) was dissolved in acetonitrile (40 mL), then K_2_CO_3_ (20.63 g, 149.25 mmol) was added to the solution. The reaction mixture was stirred for 5 min at room temperature, then 2-bromoethanol (17.93 g, 143.51 mmol) was added to the suspension. The solution was stirred for 3 h under reflux, the reaction mixture was filtered, and the filtrate was concentrated in vacuo. The resulting product was distilled under reduced pressure. The total yield was 63%. The substance was obtained as a colorless liquid.

R_f_ (SiO_2_, ethyl acetate/methanol = 5:1) = 0.44

^1^H NMR spectrum of compound **2** (300 MHz, DMSO-d6) δ, ppm: 4.40 (t, J = 5.4 Hz, 1H, -OH); 3.54 (m, 4H, 2 × CH_2_); 3.48 (q, J = 6.2 Hz, 2H, CH_2_); 2.35 (m, 6H, 3 × CH_2_).

^13^C NMR spectrum of compound **2** (75 MHz, D_2_O) δ, ppm: 66.3; 59.5; 58.1; 53.0.

HRMS (FTMS + cESI) m/z: [M+H]^+^, calculated for (C_6_H_14_NO_2_)^+^ 132.1020, found 132.1021

#### 3.3.2. Synthesis of N-(2-Chloroethyl)-Morpholine Hydrochloride (**3**)

*N-*(2-Ethanol)-morpholine (**2**) (1.0 g, 6.71 mmol) was dissolved in chloroform (10 mL) at 0–5 °C. A solution of thionyl chloride (1.20 g, 10.09 mmol) in chloroform (5 mL) was added with stirring to the resulting solution over 15 min. The reaction mixture was stirred for 4 h under reflux, and the product was concentrated in vacuo. The residue was dissolved in ethanol, and the resulting solution was poured into 50 mL of cold diethyl ether. The white crystals that precipitated were filtered off and dried in vacuo. The total yield was 83%.

R_f_ (SiO_2_, ethyl acetate/methanol = 5:1) = 0.70

^1^H NMR spectrum of compound **3** (300 MHz, DMSO-d6) δ, ppm: 11.85 (s, 1H, •HCl); 4.07 (t, J = 6.9 Hz, 2H, CH_2_); 3.85 (m, 4H, 2 × CH_2_); 3.48 (m, 4H, 2 × CH_2_); 3.15 (m, 2H, CH_2_).

^13^C NMR spectrum of compound **3** (75 MHz, D_2_O) δ, ppm: 63.6; 58.0; 51.9; 36.8.

HRMS (FTMS + cESI) m/z: [M+H]^+^, calculated for (C_6_H_14_ClNO)^+^ 150.0681, found 150.0682 (100%), 152.0655 (40%)

#### 3.3.3. Synthesis of 4-Hydroxyazobenzene (**5**)

Aniline (**4**) (1.00 g, 10.74 mmol) was added to the aqueous solution of hydrochloric acid (18%, 8 mL) at 0–5 °C. The resulting mixture was stirred for 5 min. Then sodium nitrite (0.74 g, 10.74 mmol) in water (3 mL) was added, and the mixture was stirred for 30 min with cooling. Phenol (1.40 g, 14.88 mmol) was dissolved in 3.5N NaOH (10 mL), after which the mixture was added to a solution of a diazonium salt. The reaction mixture was stirred for 1 h at 0–5 °C. The brown precipitate that formed was filtered off, dried and purified by column chromatography (SiO_2_, hexane/ethyl acetate = 5:1). The total yield was 71%. Compound **5** was obtained as yellow-orange crystals.

R_f_ (SiO_2_, hexane/ethyl acetate = 5:1) = 0.70

^1^H NMR spectrum of compound **5** (300 MHz, DMSO-d6) δ, ppm: 10.31 (s, 1H, -OH); 7.81 (m, 4H, 4 × CH); 7.52 (m, 3H, 3 × CH); 6.95 (m, 2H, 2 × CH)

^13^C NMR spectrum of compound **5** (75 MHz, DMSO-d6) δ, ppm: 161.0; 152.1; 145.2; 130.5; 129.4; 124.9; 122.1; 116.0.

HRMS (FTMS + cESI) m/z: [M+H]^+^, calculated for (C_12_H_11_N_2_O)^+^ 199.0871, found 199.0866

HPLC retention time: 8.95 min

#### 3.3.4. Synthesis of 4-(2-(N-Morpholino)-Ethoxy)-Azobenzene (**6**)

K_2_CO_3_ (349 mg, 2.53 mmol), *N-*(2-chloroethyl)-morpholine hydrochloride (3) (282 mg, 2.52 mmol) and KI (168 mg, 1.01 mmol) were added to a solution of 4-hydroxyazobenzene (**5**) (200 mg, 1.01 mmol) in acetonitrile (6 mL). The reaction mixture was stirred for 8 h under reflux. The progress of the reaction was monitored by TLC. After separation of the precipitate, the filtrate was extracted with ethyl acetate from water. The organic phase was washed with brine and dried with Na_2_SO_4_, then the solvent was removed in vacuo. The product was purified by column chromatography (SiO_2_, ethyl acetate/methanol = 5:1) and concentrated in vacuo. The total yield was 85%. The substance was obtained as orange crystals. Purity by HPLC ≥ 95%.

R_f_ (SiO_2_, ethyl acetate/methanol = 5:1) = 0.56

^1^H NMR spectrum of compound *E*-**6** (600 MHz, CDCl_3_) δ, ppm: 7.91 (m, 2H, 2 × CH, J = 9.0 Hz); 7.88 (m, 2H, 2 × CH); 7.50 (m, 2H, 2 × CH); 7.44 (m, 1H, 1 × CH); 7.02 (m, 2H, 2 × CH); 4.22 (t, J = 5.7 Hz, 2H, 1 × CH_2_); 3.76 (t, J = 4.7 Hz, 4H, 2 × CH_2_); 2.86 (t, J = 5.7 Hz, 2H, 1 × CH_2_); 2.63 (m, 4H, 2 × CH_2_).

^13^C NMR spectrum of compound *E*-6 (150 MHz, CDCl_3_) δ, ppm: 161.27; 152.93; 147.32; 130.55; 129.18; 124.89; 122.72; 114.98; 66.99; 66.26; 57.69; 54.25.

^1^H NMR spectrum of compound *Z*-**6** (600 MHz, CDCl_3_) δ, ppm: 7.28 (m, 2H, 2 × CH); 7.16 (m, 1H, 1 × CH); 6.89 (m, 2H, 2 × CH, J = 9.0 Hz); 6.85 (m, 2H, 2 × CH); 6.75 (m, 2H, 2 × CH); 4.08 (t, J = 5.7 Hz, 2H, 1 × CH_2_); 3.73 (t, J = 4.7 Hz, 4H, 2 × CH_2_); 2.79 (t, J = 5.7 Hz, 2H, 1 × CH_2_); 2.58 (m, 4H, 2 × CH_2_).

^13^C NMR spectrum of compound *Z*-**6** (150 MHz, CDCl_3_) δ, ppm: 161.57; 152.78; 147.17; 129.09; 123.82; 119.96; 115.03; 114.44; 66.99; 66.26; 57.69; 54.25.

HRMS (FTMS + cESI) m/z: [M+H]^+^, calculated for (C_18_H_22_N_3_O_2_)^+^ 312.1707, found 312.1709

HPLC retention time: 2.70 min (*Z*-form of **6**); 4.75 min (*E*-form of **6**).

#### 3.3.5. Preparation of a Micellar Solution of 4-(2-(N-Morpholino)-Ethoxy)-Azobenzene (**6**)

A solution of 4-(2-(*N*-morpholino)-ethoxy)-azobenzene (**6**) (12.0 mg) in dichloromethane (1 mL) was added dropwise to a freshly prepared 4% aqueous solution of Kolliphor ^®^ ELP (2 mL) heated to 45 °C with continuous argon bubbling. Bubbling was continued until strong foaming started. A clear orange solution with a concentration of 0.6% was obtained, which was subsequently filtered in turn through a 0.45-µm PTFE filter and then through a 0.22-µm PTFE filter.

#### 3.3.6. Measurement of the Hydrodynamic Size of Ethercaine Micelles Using Dynamic Light Scattering (DLS) Method

DLS measurements were performed on a Malvern Zetasizer Nano ZS series device (Malvern Panalytical, Malvern, United Kingdom) with a He–Ne laser (633 nm). The measurement took place in a 12 mm square disposable polystyrene cuvette at 25 °C. A 0.6% micellar solution of ethercaine was diluted with 4% aqueous solution of Kolliphor ^®^ ELP to concentrations suitable for DLS measurements. All the micellar test solutions were kept in the dark for at least 24 h before the analysis. For *Z*-form measurements, the solution in the cuvette was irradiated with UV light (λ = 395 nm) for 30 s, placed in the device, and measurements were started immediately. All manipulations were performed in the darkened room. Zetasizer software (Malvern Panalytical, Malvern, UK) was used for data curation and representation.

### 3.4. Biology

All manipulations with animals were approved by the Committee of National Medical Research Radiological Centre of the Ministry of Health of the Russian Federation for bioethical control over the maintenance and use of laboratory animals for scientific purposes (Minutes №14 dated 3 March 2021) and performed in accordance with the national and international rules for the humane treatment of animals (European Convention for the Protection of Vertebrate Animals Used for Experimental and Other Scientific Purposes, Council of Europe (ETS 123), Eighth Edition of the Guide for the Care and Use of Laboratory Animals (NRC 2011)) [51]. All materials, methods, and experimental procedures where animals were used are described in accordance with ARRIVE rules [52].

#### 3.4.1. Preparation of Animals and Arrangement of Groups

Four animals with a baseline sensitivity threshold of 1–2 touches with the anesthesiometer’s filament to the eye until a closure of the eyelids were selected for further experiments. These animals were used in the experiments repeatedly with an interval of at least 7 days between the experiments. Each eye was considered as a separate experimental point. Two test groups (the test compound with and without irradiation), two positive control groups (with and without irradiation), and two negative control groups (with and without irradiation) were used in the experiment. A 4% aqueous solution of Kolliphor ^®^ ELP was chosen as a negative control, while a 2% lidocaine solution (Lidocaine, 2% injection solution, Borisov Plant of Medical Drugs, Borisov, Republic of Belarus) served as a positive control. Four animals were used in the experimental groups and in the positive control groups (*n* = 8). Two animals per negative control group (*n* = 4) were used due to the expected lack of effect and to minimize the number of animals in the experiment. Animals were randomly chosen for the negative control group. Each animal was used only once within the same group. Before an experiment, the animals were immobilized using the fixing bag (VetNode bag No. 3, VetNode, Moscow, Russia) leaving the head free. Before each experiment, the animals’ eyelashes were cut out and the basic threshold of sensitivity of the eye cornea to tactile contact was determined using an anesthesiometer.

#### 3.4.2. Irradiation Modes

Experiments with animals were carried out in a dark room with totally shaded windows. Since experiments cannot be performed in total darkness, a 50 W LED light source with a wavelength of 625 nm, which lies outside the absorption bands of the UV/Vis spectrum of the test compound, was used for illumination. It was located in the corner of the room at a distance of approx. 2 m from the animal and turned away from the animal and the experimenter in order to avoid direct exposure to bright light on the eyes. In some of the experiments, where it is stated, rabbit eyes were irradiated with a wavelength of 395 nm (LED flashlight, 3 W) or conventional white light (λ = 420–700 nm, LED flashlight, 0.5 W). Irradiation was carried out from a distance of about 10 cm from the eyes. Irradiation duration was 30 s unless otherwise noted. Before the start of the experiments, a test solution was kept in the dark for at least 24 h. Before the test solution instillation in a conjunctival sac during the experiments with UV-light exposure, test solutions were preliminarily irradiated for 5 min with light at a wavelength of 395 nm (LED flashlight, 0.5 W). In the experiments with UV irradiation, the instillation of the test solution was also performed under 395 nm irradiation; in addition, in these experiments, before each measurement, the test eye was irradiated with 395 nm UV light for 30 s.

#### 3.4.3. Determination of the Rabbit Eye’s Cornea Sensitivity Using the Surface Anesthesia Method

This method was adapted from Regnier method [49]. After immobilizing an animal in the fixing bag and cutting off the eyelashes, the animal was allowed to adapt to the darkness in the room for 10 min. Before the drug administration, the conjunctival sac was pulled back with the help of the thumb and forefinger while pinching the lacrimal canal with the middle finger. The test solution (0.2 mL) was instilled into a conjunctival sac, after which the conjunctival sac was held for 30 s with the help of fingers. The same procedure was carried out with the second eye (this experiment can be performed both using one or two eyes in parallel). After the instillation, the sensitivity threshold of the eye’s cornea to tactile impact was determined for each eye with the anesthesiometer. The first determination was performed at the 8th minute from the moment of the instillation and then at the 10th, 12th, 15th, 25th, 30th, 35th, 40th, 45th, 50th, 55th and 60th mins (13 values overall for each eye). Each time, the minimum number (but not more than 100) of the anesthesiometer’s filament touches with the same strength and rhythm, causing closure of the eyelids was recorded. The Regnier index was calculated for each eye as a total of 13 values using the formula (Equation (1)):

(1) $$\mathrm{Regnier}\;\mathrm{index}\;\left(\mathrm{RI}\right)=\;\sum_{i=1}^{13}n_{i}$$

where *n* is the number of touches before the eyelids closed. The experiment was repeated for each animal in the group (two animals (*n* = 4) for the negative control group and four animals (*n* = 8) for other groups).

#### 3.4.4. Determination of the Rabbit Eye’s Cornea Sensitivity Using the Surface Anesthesia Model during Multiple Reversible Photoswitching

The experiment was carried out according to the method described above using the adapted Regnier method. Due to the need for frequent exposure to different light sources, experiments with multiple reversible photoswitching were not performed with two eyes in parallel (i.e., only one eye was used in each experiment). Before the instillation, the test substance solution was irradiated with 395 nm light for 5 min, after which, before each measurement, the eye was irradiated with the corresponding (395 nm UV or white) light for 30 s (for more details on irradiation modes, see the corresponding subsection). The experiment was repeated 8 times using four animals.

#### 3.4.5. Determination of the Local Irritating Effect

Simultaneously with the local anesthetic activity determination, the local irritating effect of the test compound was determined by visual observation of test compound’s effects on the eyes during the experiment. The key parameters for estimating the negative impact included the hyperemia and induration of the conjunctiva, nictitating membrane, edges and tissues of eyelids, and sclera. The local irritating effect was estimated with each administration of the test and control compounds throughout the entire experiment (0–60 min from the instant of administration) in 5 min increments, and additionally 24 h after the end of the study.

## 4. Conclusions

In this work, we obtained a new photoswitchable derivative of fotocaine, a known photoswitchable blocker of potential-dependent sodium channels. We named it ethercaine. The resulting compound is an ether of 4-hydroxyazobenzene and *N*-(2-hydroxyethyl)-morpholine, which was synthesized by a simple scheme and has enhanced spectral characteristics. Ethercaine was studied in vivo using a surface anesthesia model in rabbits and was shown to have photoswitchable local anesthetic properties. The mean values of the Regnier index for the *E*- and *Z*-forms differ by one order of magnitude, which illustrates that biological activity clearly depends on the presence or absence of light exposure. A micellar solution of ethercaine (**6**) has no local irritating effect. The results obtained show that it is promising to use compounds of this class for the development of new photoswitchable agents with improved solubility in water and optimal photobiological properties for clinical use.

## Figures and Tables

**Table 1:** Studies of the local anesthetic effect of ethercaine (6) in vivo.

Compound Under Study	Regnier Index (min—13, max—1300)	
	In the Dark	Under Irradiation (λ = 395 nm)
Lidocaine 2%	451 ± 40 (*n* = 8) *	469 ± 37 (*n* = 8)
Ethercaine (6) (0.6% solution in 4% Kolliphor ^®^ ELP)	232 ± 50 (*n* = 8)	22 ± 3 (*n* = 8)
4% Kolliphor ^®^ ELP	13 (*n* = 4)	13 (*n* = 4)

* Note: All data are expressed as the mean ± standard deviation.

## Data Availability

The data presented in this study are available from the authors on request.

## Supplementary Materials

The following supporting information can be downloaded at: https://www.mdpi.com/article/10.3390/ijms23105352/s1

## References

[1] Daubländer M., Müller R., Lipp M.D. (1997). The Incidence of Complications Associated with Local Anesthesia in Dentistry. Anesth. Prog..

[2] Keskin Yalcin B. (2020). Complications Associated with Local Anesthesia in Oral and Maxillofacial Surgery. Topics in Local Anesthetics.

[3] Bezerra M.M., Leão R.A.C., Miranda L.S.M., De Souza R.O.M.A. (2020). A Brief History behind the Most Used Local Anesthetics. Tetrahedron.

[4] McCaughey W. (1992). Adverse Effects of Local Anaesthetics. Drug Saf..

[5] Zink W., Graf B.M. (2004). Local Anesthetic Myotoxicity. Reg. Anesth. Pain Med..

[6] Wolfe J.W., Butterworth J.F. (2011). Local Anesthetic Systemic Toxicity: Update on Mechanisms and Treatment. Curr. Opin. Anaesthesiol..

[7] Sekimoto K., Tobe M., Saito S. (2017). Local Anesthetic Toxicity: Acute and Chronic Management. Acute Med. Surg..

[8] Eggleston S.T., Lush L.W. (1996). Adverse Reactions Aromatic—Intermediate Chain—Amine Salt. Ann. Pharmacother..

[9] Bourne E., Wright C., Royse C. (2010). A Review of Local Anesthetic Cardiotoxicity and Treatment with Lipid Emulsion. Local Reg. Anesth..

[10] Bhansali D., Teng S.L., Lee C.S., Schmidt B.L., Bunnett N.W., Leong K.W. (2021). Nanotechnology for Pain Management: Current and Future Therapeutic Interventions. Nano Today.

[11] He Y., Qin L., Huang Y., Ma C. (2020). Advances of Nano-Structured Extended-Release Local Anesthetics. Nanoscale Res. Lett..

[12] Prabhakar A., Ward C.T., Watson M., Sanford J., Fiza B., Moll V., Kaye R.J., Morgan Hall O., Cornett E.M., Urman R.D. (2019). Liposomal Bupivacaine and Novel Local Anesthetic Formulations. Best Pract. Res. Clin. Anaesthesiol..

[13] Mendoza G., Arruebo M. (2020). Light-Triggered Nanoparticles for Pain Management. Expert Opin. Drug Deliv..

[14] Filonenko E.V. (2022). Clinical Implementation and Scientific Development of Photodynamic Therapy in Russia in 2010–2020. Biomed. Photonics.

[15] Pang Q., Zhao J., Zhang S., Zhang X. (2020). Near-Infrared Triggered on-Demand Local Anesthesia Using a Jammed Microgels System. J. Biomater. Sci. Polym. Ed..

[16] Rwei A.Y., Wang B.Y., Ji T., Zhan C., Kohane D.S. (2017). Enhanced Triggering of Local Anesthetic Particles by Photosensitization and Photothermal Effect Using a Common Wavelength. Nano Lett..

[17] Chen M.C., Chan H.A., Ling M.H., Su L.C. (2017). Implantable Polymeric Microneedles with Phototriggerable Properties as a Patient-Controlled Transdermal Analgesia System. J. Mater. Chem. B.

[18] Weiser B.P., Kelz M.B., Eckenhoff R.G. (2013). In Vivo Activation of Azipropofol Prolongs Anesthesia and Reveals Synaptic Targets. J. Biol. Chem..

[19] Shalabi A.R., Yu Z., Zhou X., Jounaidi Y., Chen H., Dai J., Kent D.E., Feng H.-J., Forman S.A., Cohen J.B. (2020). A Potent Photoreactive General Anesthetic with Novel Binding Site Selectivity for GABAA Receptors. Eur. J. Med. Chem..

[20] Zhang W., Ji T., Li Y., Zheng Y., Mehta M., Zhao C., Liu A., Kohane D.S. (2020). Light-Triggered Release of Conventional Local Anesthetics from a Macromolecular Prodrug for on-Demand Local Anesthesia. Nat. Commun..

[21] Font J., López-Cano M., Notartomaso S., Scarselli P., Di Pietro P., Bresolí-Obach R., Battaglia G., Malhaire F., Rovira X., Catena J. (2017). Optical Control of Pain in Vivo with a Photoactive MGlu5 Receptor Negative Allosteric Modulator. Elife.

[22] López-Cano M., Font J., Aso E., Sahlholm K., Cabré G., Giraldo J., De Koninck Y., Hernando J., Llebaria A., Fernández-Dueñas V. (2021). Remote Local Photoactivation of Morphine Produces Analgesia without Opioid-related Adverse Effects. Br. J. Pharmacol..

[23] Wang F., Bélanger E., Paquet M.E., Côté D.C., De Koninck Y. (2016). Probing Pain Pathways with Light. Neuroscience.

[24] Gazerani P. (2017). Shedding Light on Photo-Switchable Analgesics for Pain. Pain Manag..

[25] Kienzler M.A., Isacoff E.Y. (2017). Precise Modulation of Neuronal Activity with Synthetic Photoswitchable Ligands. Curr. Opin. Neurobiol..

[26] Jarrin S., Finn D.P. (2019). Optogenetics and Its Application in Pain and Anxiety Research. Neurosci. Biobehav. Rev..

[27] Beaudry H., Daou I., Ribeiro-da-Silva A., Séguéla P. (2016). Can We Use Optogenetics to Treat Chronic Pain?. Douleur Analg..

[28] Deisseroth K. (2011). Optogenetics. Nat. Methods.

[29] Bonin R.P., Wang F., Desrochers-Couture M., Ga¸secka A., Boulanger M.-E., Côté D.C., De Koninck Y. (2016). Epidural Optogenetics for Controlled Analgesia. Mol. Pain.

[30] Broichhagen J., Frank J.A., Trauner D. (2015). A Roadmap to Success in Photopharmacology. Acc. Chem. Res..

[31] Gómez-Santacana X., Pittolo S., Rovira X., Lopez M., Zussy C., Dalton J.A.R., Faucherre A., Jopling C., Pin J.P., Ciruela F. (2017). Illuminating Phenylazopyridines to Photoswitch Metabotropic Glutamate Receptors: From the Flask to the Animals. ACS Cent. Sci..

[32] Pittolo S., Gómez-Santacana X., Eckelt K., Rovira X., Dalton J., Goudet C., Pin J.-P., Llobet A., Giraldo J., Llebaria A. (2014). An Allosteric Modulator to Control Endogenous G Protein-Coupled Receptors with Light. Nat. Chem. Biol..

[33] Rovira X., Trapero A., Pittolo S., Zussy C., Faucherre A., Jopling C., Giraldo J., Pin J.P., Gorostiza P., Goudet C. (2016). OptoGluNAM4.1, a Photoswitchable Allosteric Antagonist for Real-Time Control of MGlu4 Receptor Activity. Cell Chem. Biol..

[34] Stein M., Middendorp S.J., Carta V., Pejo E., Raines D.E., Forman S.A., Sigel E., Trauner D. (2012). Azo-Propofols: Photochromic Potentiators of GABAA Receptors. Angew. Chem. Int. Ed..

[35] Schönberger M., Trauner D. (2014). A Photochromic Agonist for μ-Opioid Receptors. Angew. Chem. Int. Ed..

[36] Mostyn S.N., Sarker S., Muthuraman P., Raja A., Shimmon S., Rawling T., Cioffi C.L., Vandenberg R.J. (2020). Photoswitchable ORG25543 Congener Enables Optical Control of Glycine Transporter 2. ACS Chem. Neurosci..

[37] Frank J.A., Moroni M., Moshourab R., Sumser M., Lewin G.R., Trauner D. (2015). Photoswitchable Fatty Acids Enable Optical Control of TRPV1. Nat. Commun..

[38] Mourot A., Fehrentz T., Le Feuvre Y., Smith C.M., Herold C., Dalkara D., Nagy F., Trauner D., Kramer R.H. (2012). Rapid Optical Control of Nociception with an Ion-Channel Photoswitch. Nat. Methods.

[39] Schoenberger M., Damijonaitis A., Zhang Z., Nagel D., Trauner D. (2014). Development of a New Photochromic Ion Channel Blocker via Azologization of Fomocaine. ACS Chem. Neurosci..

[40] Vetter I., Deuis J.R., Mueller A., Israel M.R., Starobova H., Zhang A., Rash L.D., Mobli M. (2017). NaV1.7 as a Pain Target—From Gene to Pharmacology. Pharmacol. Ther..

[41] Shen H., Liu D., Wu K., Lei J., Yan N. (2019). Structures of Human Na v 1.7 Channel in Complex with Auxiliary Subunits and Animal Toxins. Science.

[42] Panigel J., Cook S.P. (2011). A Point Mutation at F1737 of the Human Na v 1.7 Sodium Channel Decreases Inhibition by Local Anesthetics. J. Neurogenet..

[43] Fozzard H.A., Sheets M.F., Hanck D.A. (2011). The Sodium Channel as a Target for Local Anesthetic Drugs. Front. Pharmacol..

[44] Noreng S., Li T., Payandeh J. (2021). Structural Pharmacology of Voltage-Gated Sodium Channels. J. Mol. Biol..

[45] Körner J., Albani S., Sudha Bhagavath Eswaran V., Roehl A.B., Rossetti G., Lampert A. (2022). Sodium Channels and Local Anesthetics—Old Friends With New Perspectives. Front. Pharmacol..

[46] Skarga V.V., Nevezhin E.V., Matrosov A.A., Negrebetsky V.V., Malakhov M. (2019). V Detection of hydroperoxides in solutions of photooxidized psoralen. Fine Chem. Technol..

[47] Beveridge D.L., Jaffé H.H. (1966). The Electronic Structure and Spectra of Cis- and Trans-Azobenzene. J. Am. Chem. Soc..

[48] Vetráková Ľ., Ladányi V., Al Anshori J., Dvořák P., Wirz J., Heger D. (2017). The Absorption Spectrum of Cis -Azobenzene. Photochem. Photobiol. Sci..

[49] Vogel H.G., Vogel H.G. (2008). Drug Discovery and Evaluation.

[50] Morris G.M., Huey R., Lindstrom W., Sanner M.F., Belew R.K., Goodsell D.S., Olson A.J. (2009). AutoDock4 and AutoDockTools4: Automated Docking with Selective Receptor Flexibility. J. Comput. Chem..

[51] Liu Y., Huang Y., Xiao Z., Ren X., Yang C. (2011). Guide for the Care and Use of Laboratory Animals.

[52] Percie du Sert N., Hurst V., Ahluwalia A., Alam S., Avey M.T., Baker M., Browne W.J., Clark A., Cuthill I.C., Dirnagl U. (2020). The ARRIVE Guidelines 2.0: Updated Guidelines for Reporting Animal Research. Exp. Physiol..

