# Supplementary material for: A Novel Photoswitchable Azobenzene-Containing Local Anesthetic Ethercaine with Light-Controlled Biological Activity In Vivo
Source: Int J Mol Sci. 2022 May 11;23(10):5352. doi: 10.3390/ijms23105352 (PMC9141926; doi:10.3390/ijms23105352)
Supplement: Supplementary file 1 [file ijms-23-05352-s001.zip › ijms-1699018-supplementary.pdf]

1. HPLC-HRMS elution and detection parameters

HPLC-HRMS analysis of compounds was performed on a Vanquish liquid chromatographic system coupled to a Q-Exactive HF-X high-resolution hybrid mass spectrometer. Separation of the sample components was performed on a reversed-phase Pyramid analytical column 75 mm long, 2 mm in inner diameter, 1.8 µm sorbent particle diameter, manufactured by Macherey-Nagel, Germany.

As component A of the mobile phase, a solution of formic acid (Fluka) in acetonitrile (Panreac, USA), and Mili Q deionized water (18.2 Ω^{-1}) in a volume ratio of 0.1/5/95 was used. A 0.1% solution of formic acid in acetonitrile (Panreac, USA) was used as component B of the mobile phase. Chromatographic separation parameters are presented in Table 1.

Table S1. HPLC separation parameters conditions.

<table>
<tr><td>Elution mode</td><td colspan="3">Gradient</td></tr>
<tr><td>Flow rate, ml/min</td><td colspan="3">0,500</td></tr>
<tr><td rowspan="6">Mobile phase composition gradient</td><td>Time. min</td><td>Component A, %</td><td>Component B, %</td></tr>
<tr><td>0,00</td><td>95</td><td>5</td></tr>
<tr><td>2,00</td><td>95</td><td>5</td></tr>
<tr><td>15,00</td><td>5</td><td>95</td></tr>
<tr><td>18,00</td><td>5</td><td>95</td></tr>
<tr><td>18,01</td><td>95</td><td>5</td></tr>
<tr><td>Column thermostat temperature, °C</td><td colspan="3">40</td></tr>
<tr><td>The volume of an aliquot of the sample applied to the column, µl</td><td colspan="3">3</td></tr>
<tr><td>Experiment time, min</td><td colspan="3">20</td></tr>
</table>

HRMS detection of compounds was carried out in Full Scan MS Positive/Negative modes during electrospray ionization at atmospheric pressure.

Parameters of the mass spectrometer ionization source are presented in Table S2, and operating parameters of the mass spectrometer modes are presented in Table S3.

Table S2. Parameters of the mass spectrometer ionization source.

№ п/п	Parameter	Parameter value
1.	Spray voltage	+/-4,0 kV
2.	Nebulizer gas flow rate	35 a.u.
3.	Auxiliary gas flow rate	15 a.u.
4.	Drying gas flow rate	5 a.u.
5.	S-lens	50 a.u.

Table S3 - Operating parameters of the mass spectrometer modes during the analysis of target compounds.

<table>
<tr><td>Mode</td><td>Parameter</td><td>Parameter value</td></tr>
<tr><td rowspan="2">Full Scan MS</td><td>Resolution</td><td>70000 a.u.</td></tr>
<tr><td>Mass range</td><td>100 – 1000 Da</td></tr>
</table>

2. HPLC-PDA elution and detection parameters

HPLC-PDA analysis of compounds was performed on a Agilent 1260 Infinity II chromatograph (Agilent Technologies, Inc., CA, USA) with 1260 DAD WR detector. Separation of the sample components was performed on a reversed-phase Hypersil Gold aQ analytical column 100 mm long, 2.1 mm in inner diameter, 1.9 µm sorbent particle diameter, manufactured by Macherey-Nagel, Germany.

As component A of the mobile phase, a solution of formic acid (Fluka) in acetonitrile (Panreac, USA), and Mili Q deionized water (18.2 Ω^{-1}) in a volume ratio of 0.1/5/95 was used. A 0.1% solution of formic acid in acetonitrile (Panreac, USA) was used as component B of the mobile phase. Chromatographic separation parameters are presented in Table 1.

Table S4. HPLC separation parameters conditions.

<table>
<tr><td>Elution mode</td><td colspan="3">Gradient</td></tr>
<tr><td>Flow rate, ml/min</td><td colspan="3">0,500</td></tr>
<tr><td rowspan="6">Mobile phase composition gradient</td><td>Time. min</td><td>Component A, %</td><td>Component B, %</td></tr>
<tr><td>0,00</td><td>100</td><td>0</td></tr>
<tr><td>1,00</td><td>100</td><td>0</td></tr>
<tr><td>10,00</td><td>5</td><td>95</td></tr>
<tr><td>12,00</td><td>5</td><td>95</td></tr>
<tr><td>12,10</td><td>100</td><td>0</td></tr>
<tr><td>Column thermostat temperature, °C</td><td colspan="3">40</td></tr>
<tr><td>The volume of an aliquot of the sample applied to the column, µl</td><td colspan="3">3</td></tr>
<tr><td>Experiment time, min</td><td colspan="3">15</td></tr>
</table>

3. NMR, HRMS and DLS data

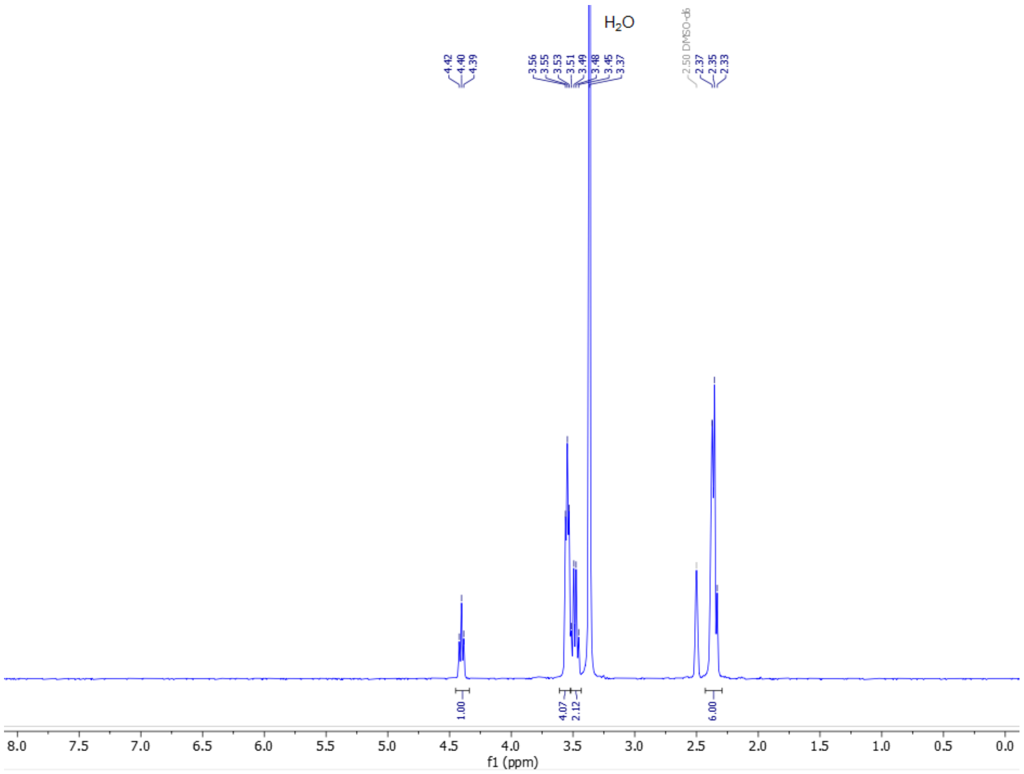

Figure S1. ^{1}H NMR (300MHz, DMSO-d6) spectrum of compound **2**.

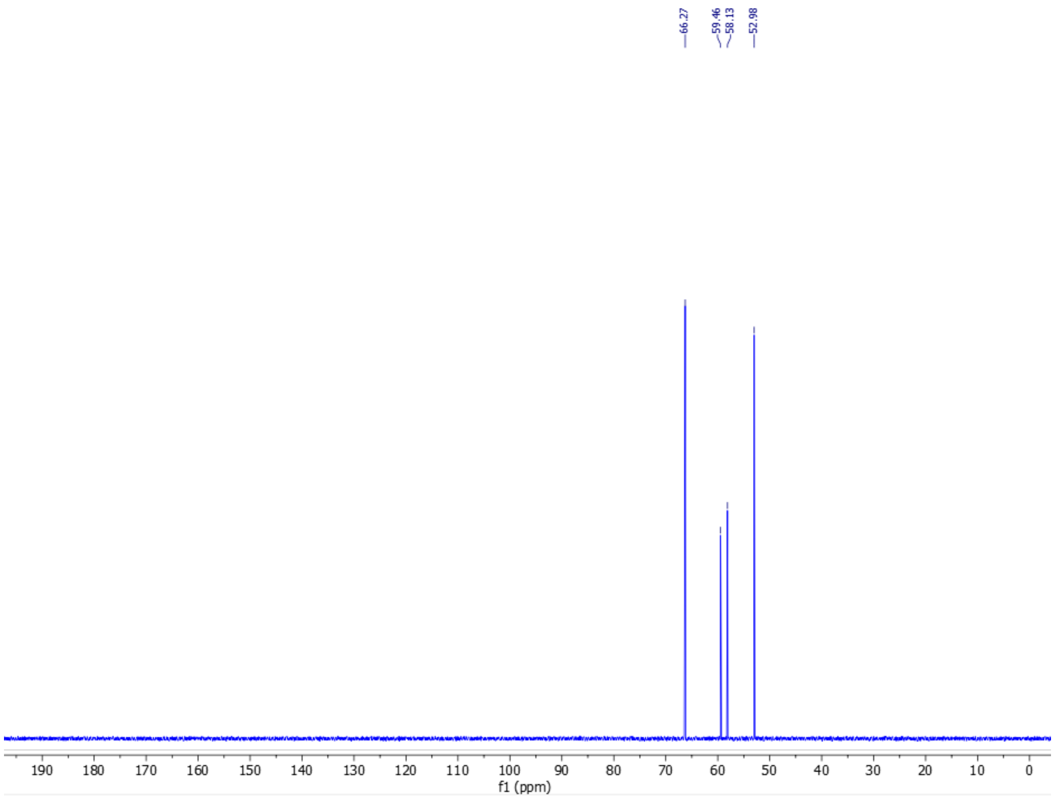

Figure S2. ^{13}C NMR (75 MHz, D_2O) spectrum of compound **2**.

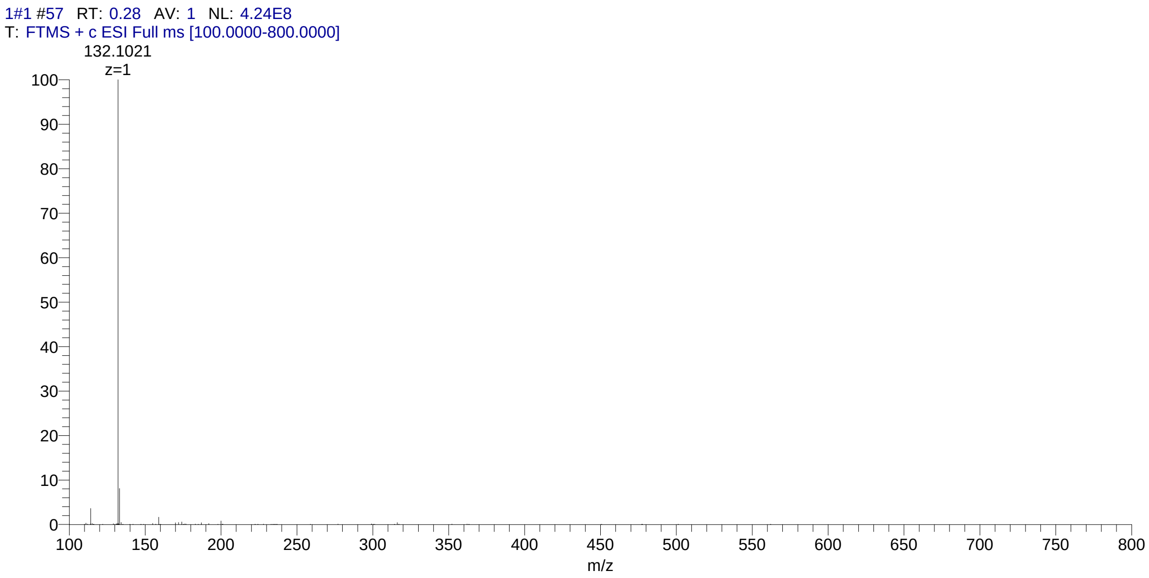

Figure S3. HRMS (FTMS + cESI) spectrum of compound **2**

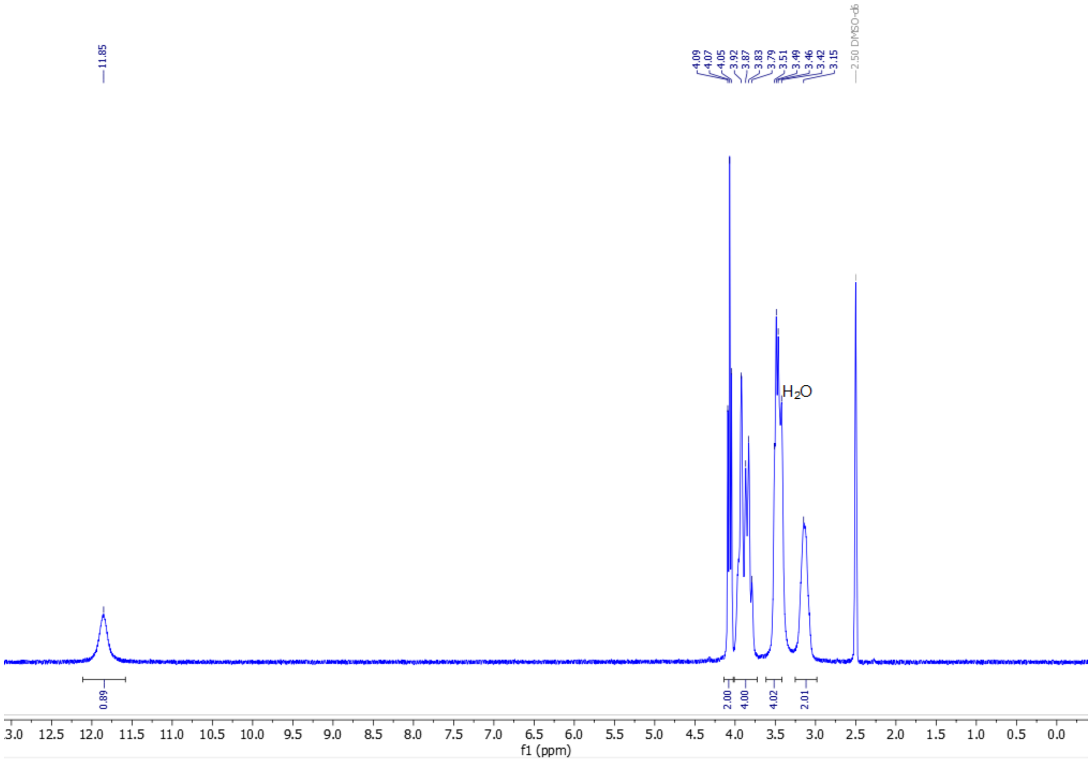

Figure S4. ^{1}H NMR (300 MHz, DMSO-d6) spectrum of compound **3**.

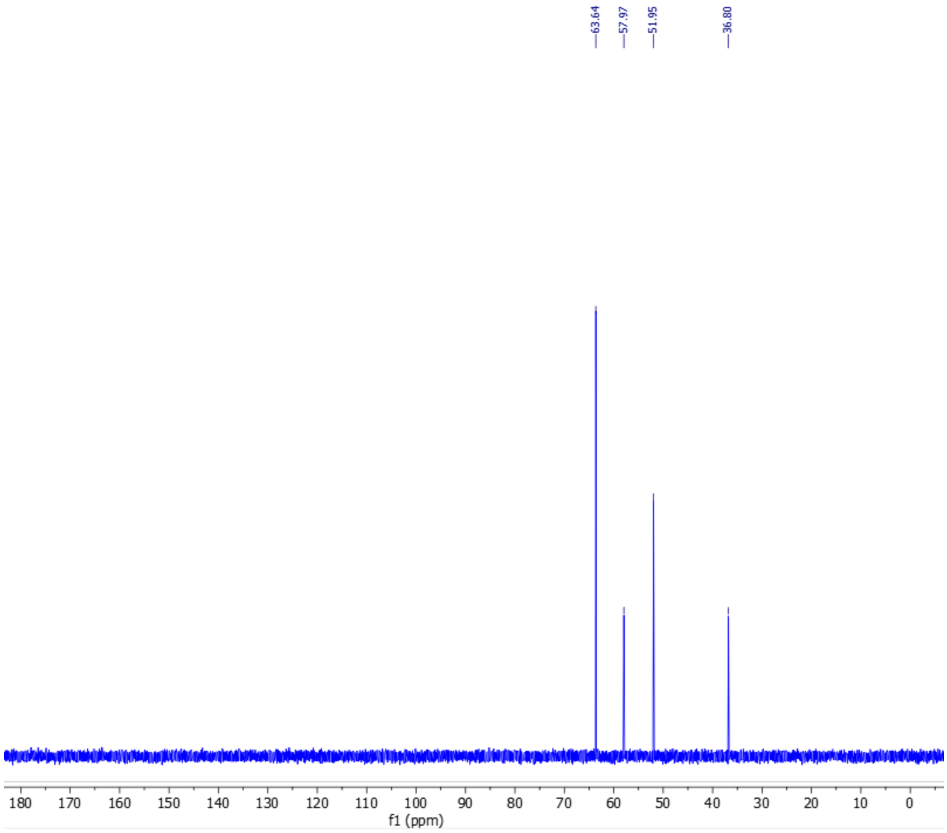

Figure S5. ^{13}C NMR (75 MHz, D_2O) spectrum of compound **3**.

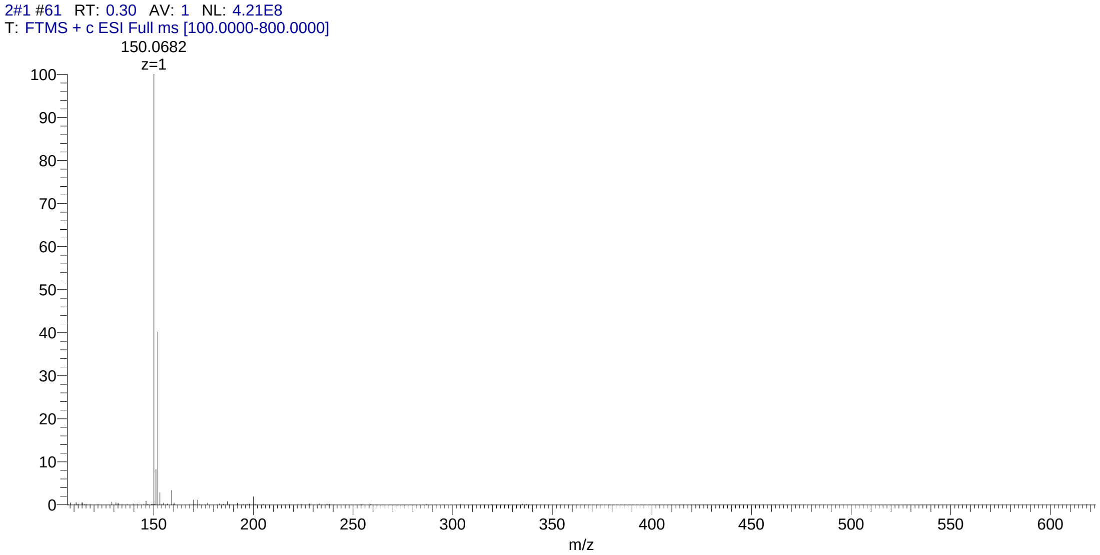

Figure S6. HRMS (FTMS + cESI) spectrum of compound **3.**

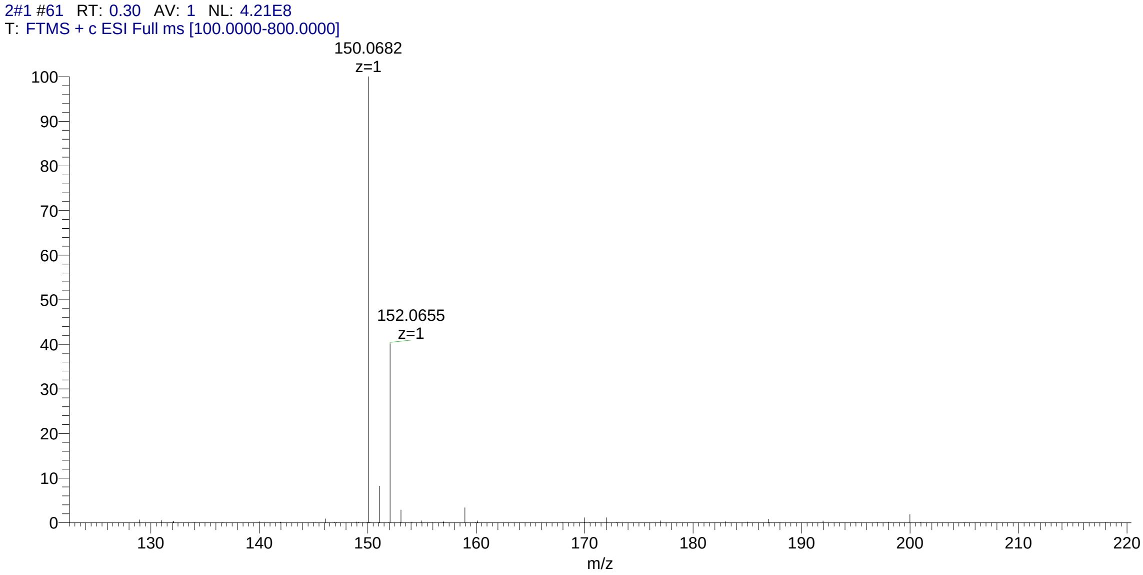

Figure S7. Fragment of HRMS (FTMS + cESI) spectrum of compound **3**. The two main peaks represent the two main stable chlorine isotopes ^{35}Cl and ^{37}Cl.

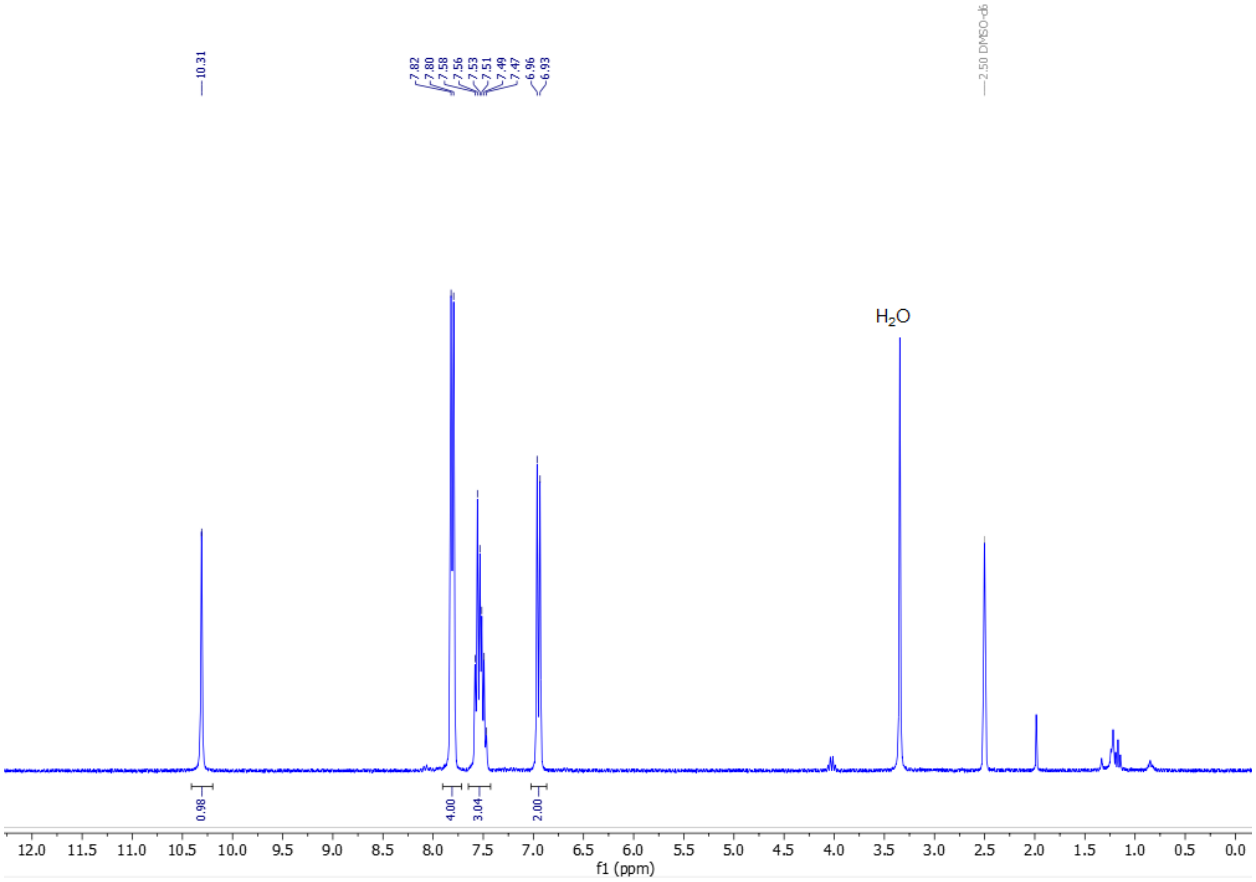

Figure S8. ^{1}H NMR (300 MHz, DMSO-d6) spectrum of compound **5**.

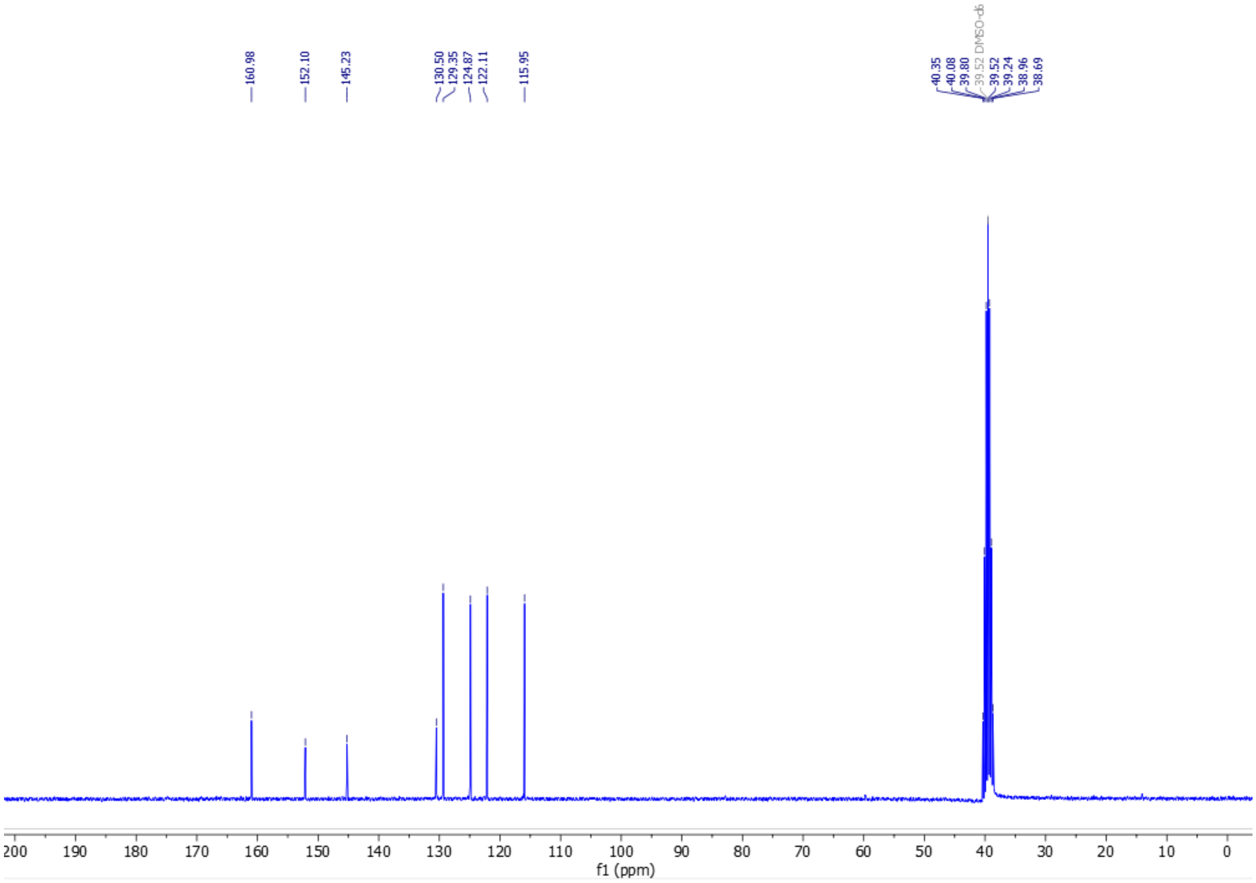

Figure S9. ^{13}C NMR (75 MHz, DMSO-d6) spectrum of compound **5**.

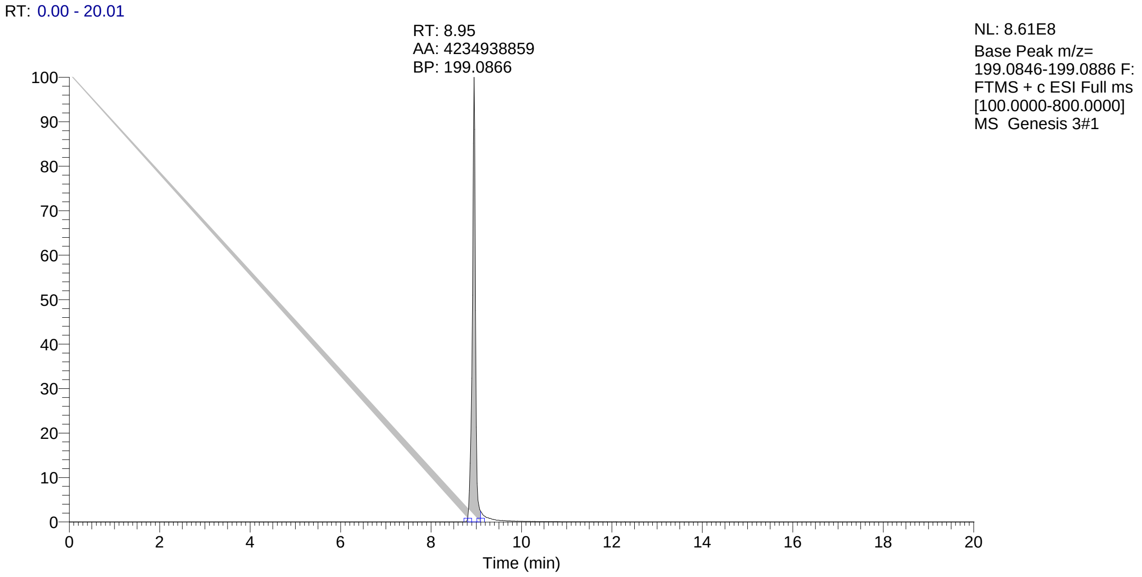

Figure S10. HPLC chromatogram of compound **5.**

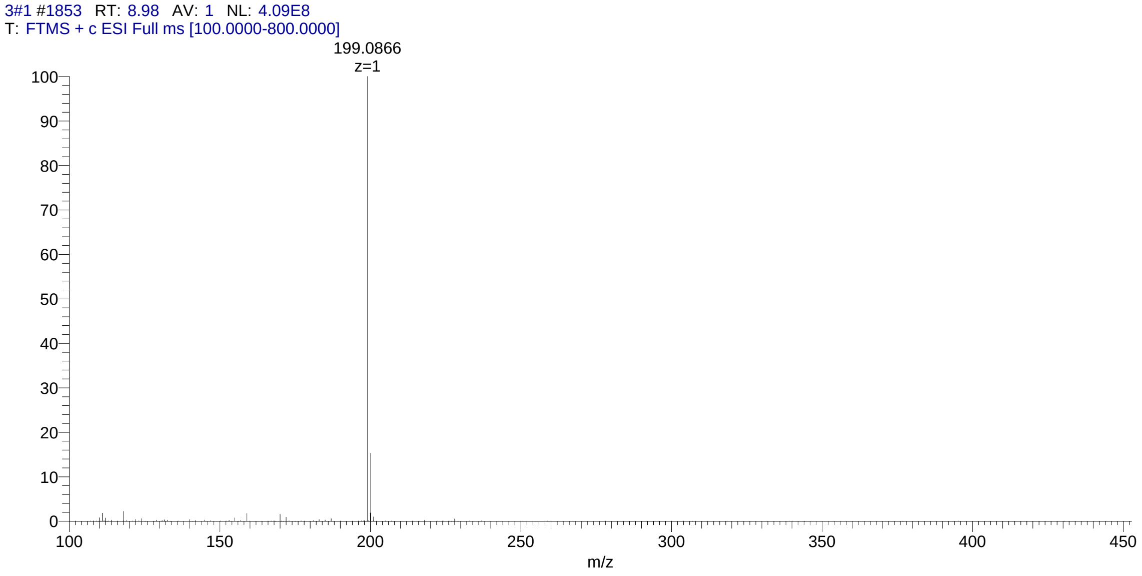

Figure S11. HRMS (FTMS + cESI) spectrum of compound **5.**

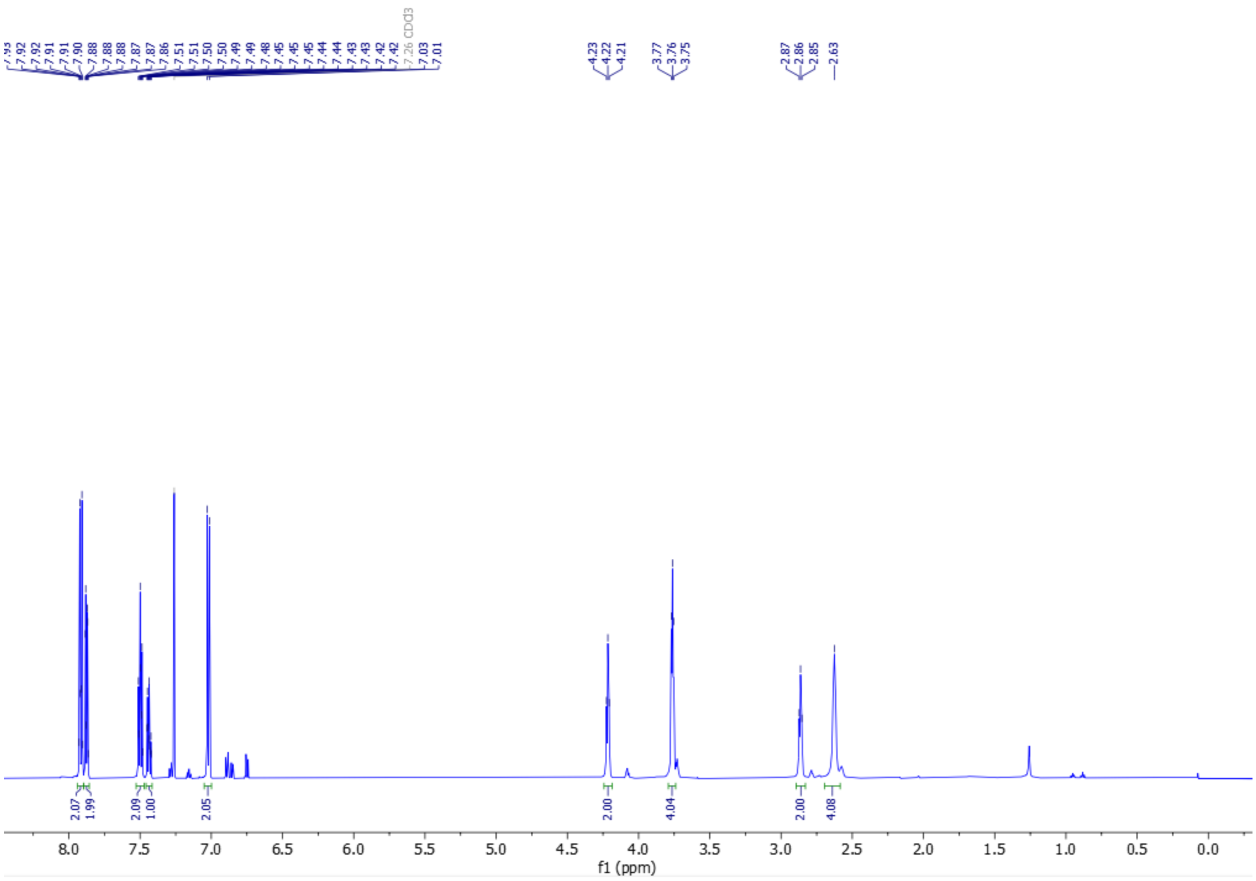

Figure S12. ^{1}H NMR (600 MHz, $CDCl_3$) spectrum of *E*-ethercaine (**6**)

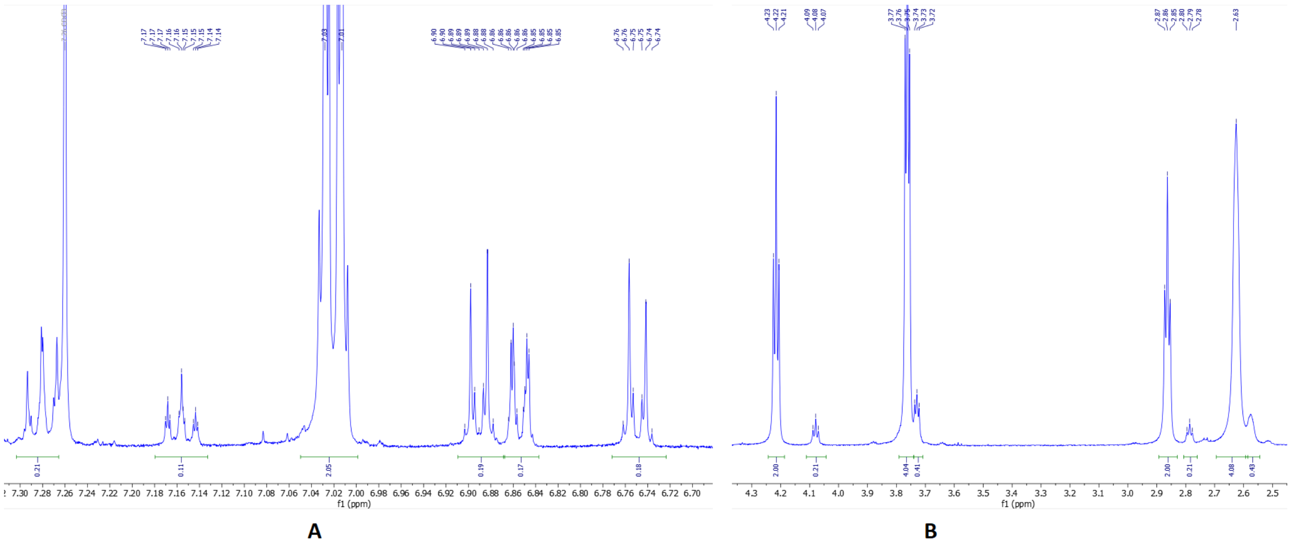

Figure S13. Fragments of aromatic (**A**) and aliphatic (**B**) regions of ^{1}H NMR (600 MHz, $CDCl_3$) spectrum of *E*-ethercaine (**6**), which demonstrates existence of minor peaks corresponding *Z*-form of ethercaine (**6**).

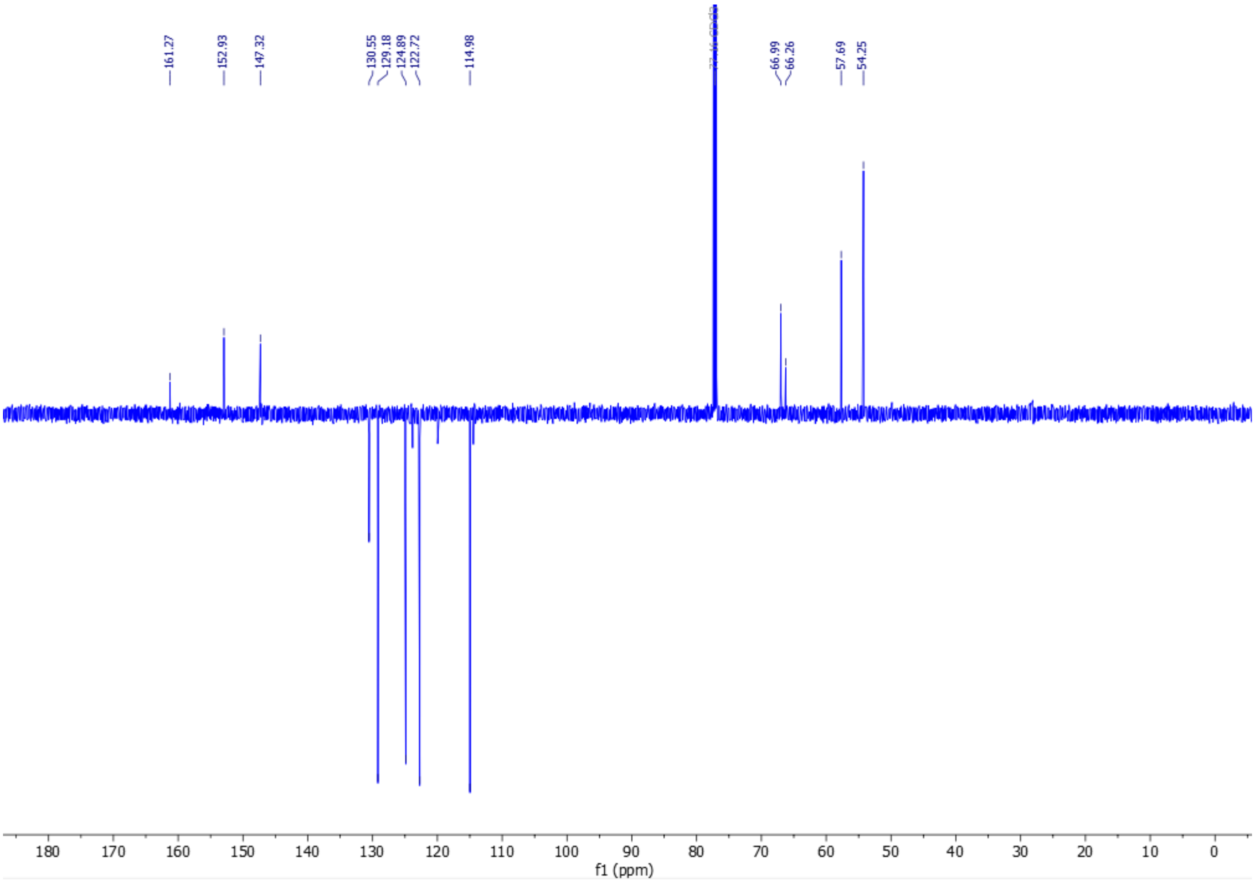

Figure S14. $^{13}C\{1H\}$ NMR (150 MHz, $CDCl_3$) spectrum of ethercaine (**6**).

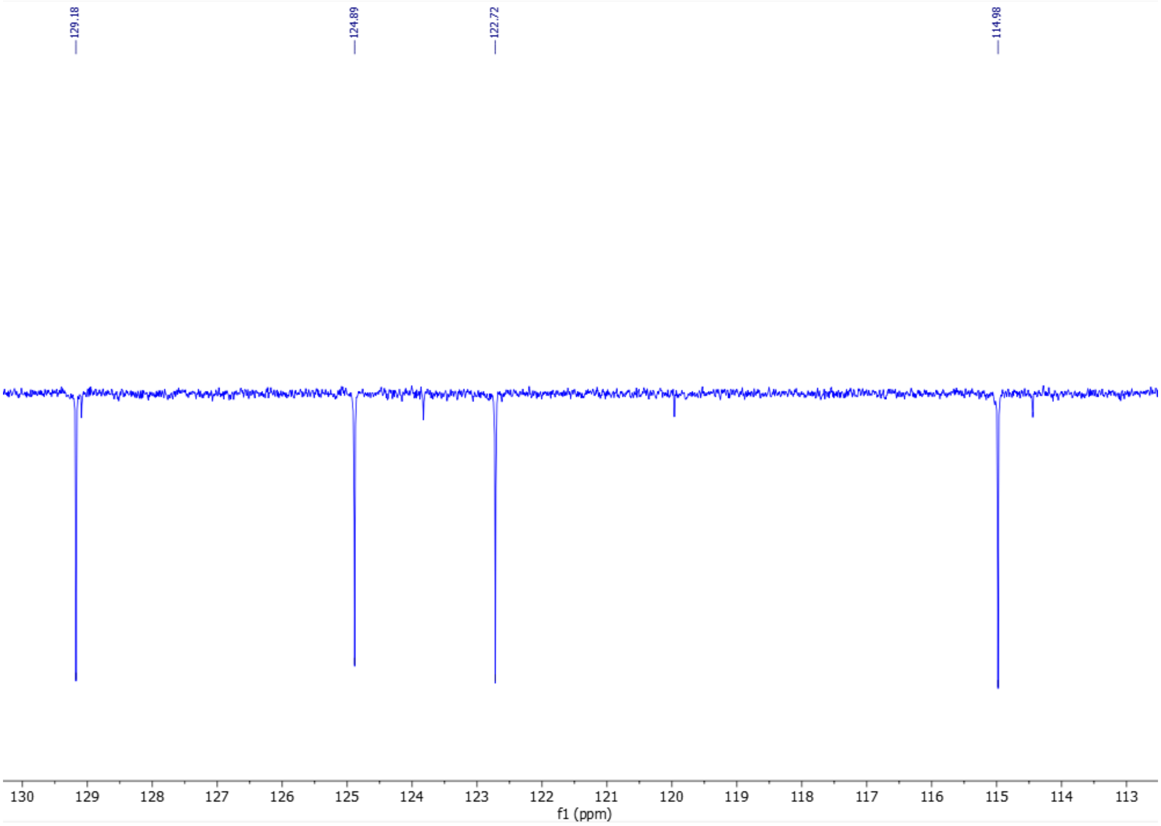

Figure S15. Fragment of $^{13}C\{1H\}$ NMR (150 MHz, $CDCl_3$) spectrum of *E*-form of ethercaine (**6**), which demonstrates existence of minor peaks corresponding *Z*-form of ethercaine (**6**).

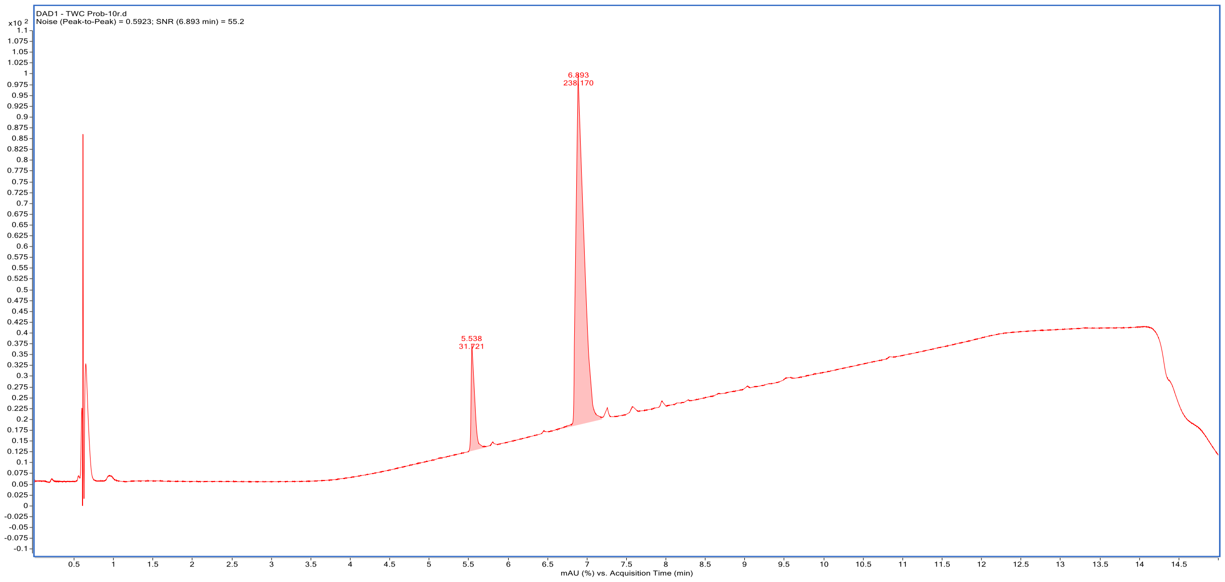

Figure S16. HPLC-PDA chromatogram of ethercaine (**6**).

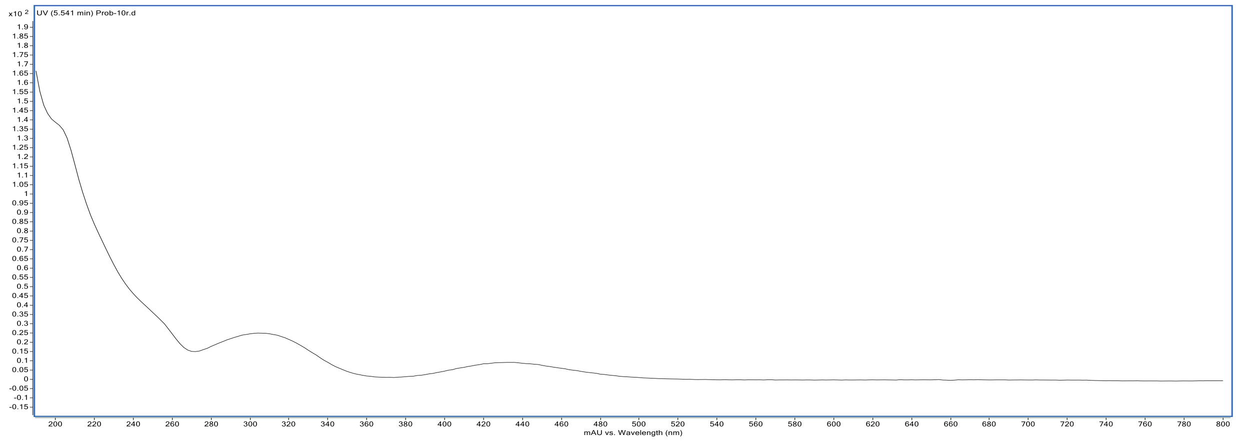

Figure S17. UV-Vis absorption spectrum of Z-ethercaine (**6**) (5.35 min peak on HPLC-PDA chromatogram, Figure S16) obtained during HPLC-PDA analysis.

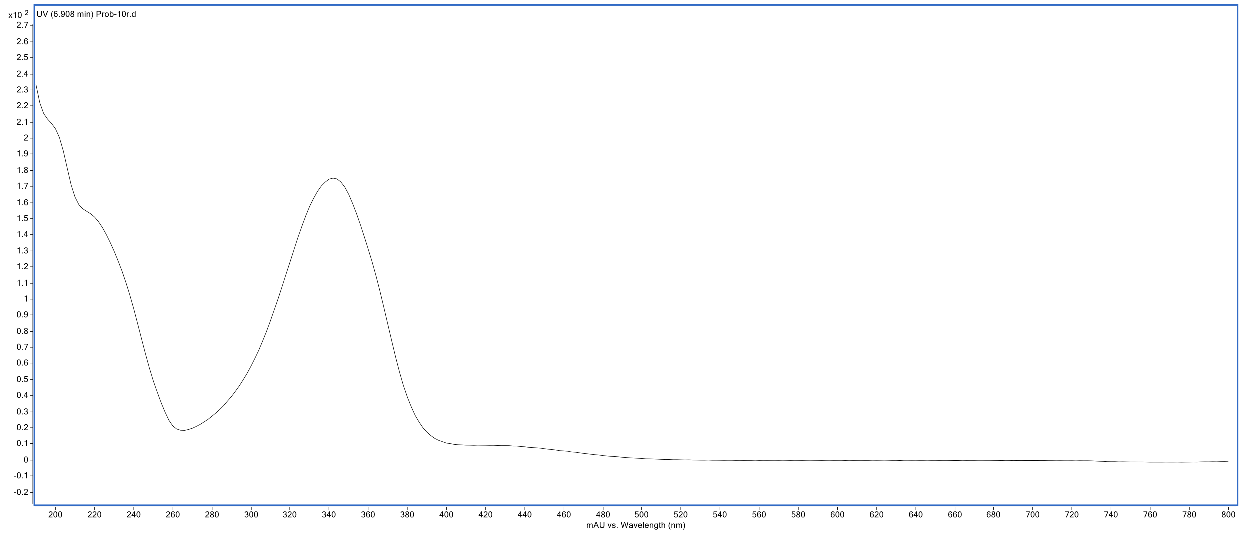

Figure S18. UV-Vis absorption spectrum of E-ethercaine (**6**) (6.89 min peak on HPLC-PDA chromatogram, Figure S16) obtained during HPLC-PDA analysis.

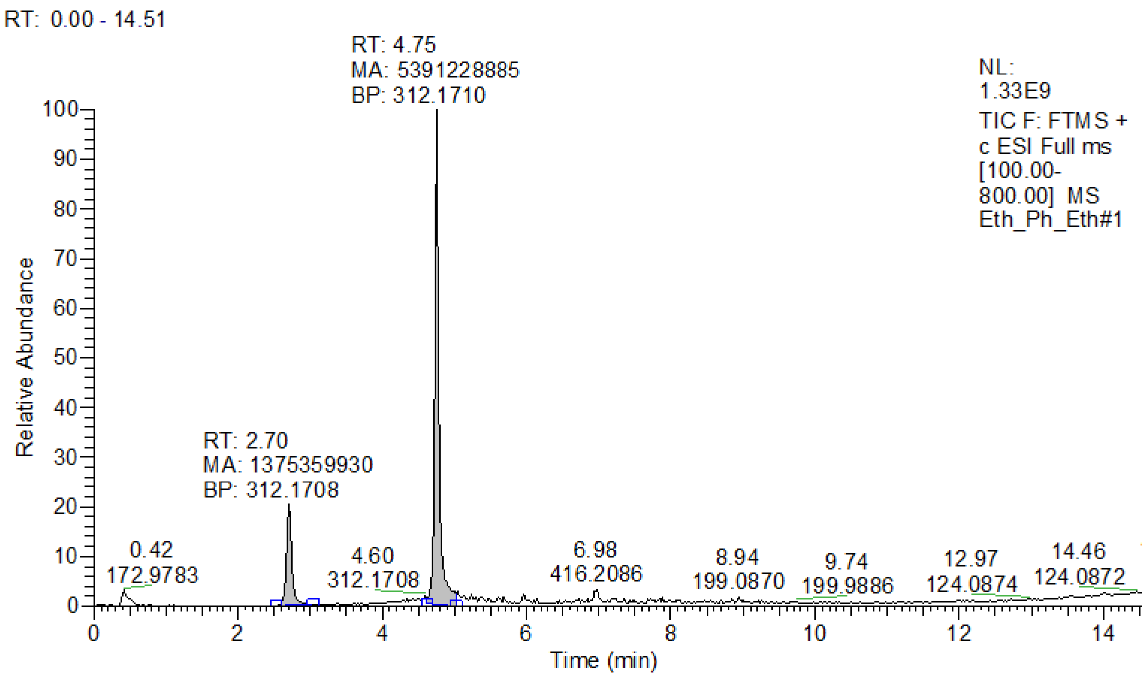

Figure S19. HPLC TIC chromatogram of ethercaine (**6**).

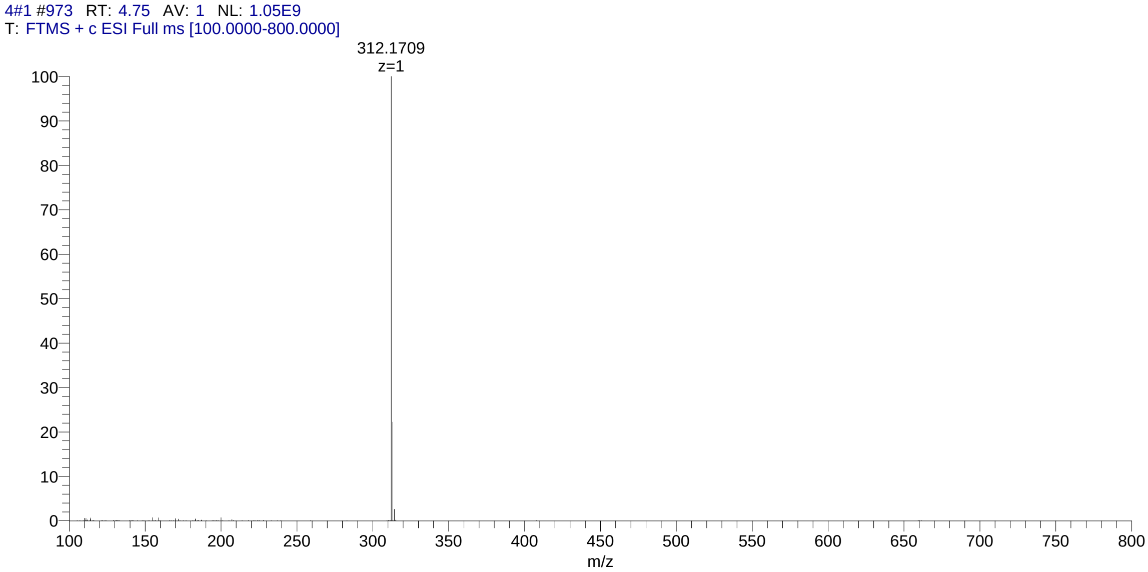

Figure S20. HRMS (FTMS + cESI) spectrum of *E*- ethercaine (**6**) (*Z*-form of ethercaine (**6**)has the identical spectrum).

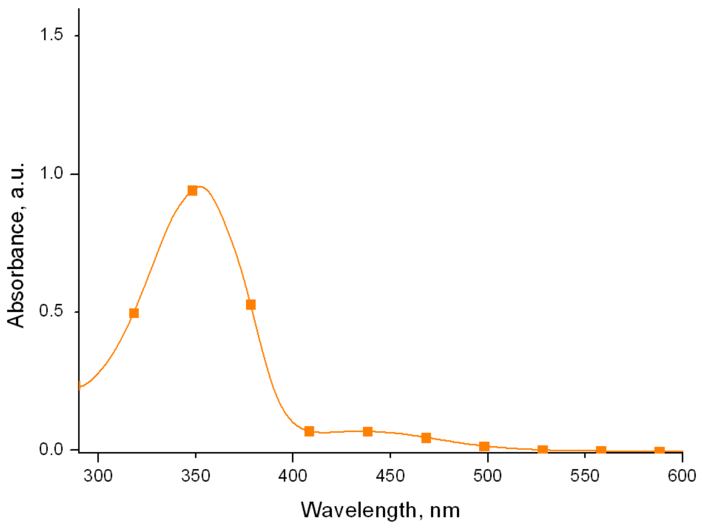

Figure S21. UV/Vis absorption spectrum of ethercaine (**6)** in DMSO.

Size Distribution Report by Volume

v2.2

Malvern

Sample Details

**Sample Name:**	foto_ether_0,6_filtr_Kolliphor 3
**SOP Name:**	mansettings.nano
**General Notes:**	

**File Name:**	151221.dts	**Dispersant Name:**	Water
**Record Number:**	6	**Dispersant RI:**	1,330
**Material RI:**	1,59	**Viscosity (cP):**	0,6527
**Material Absorbtion:**	0,010	**Measurement Date and Time:**	15 декабря 2021 г. 17:14:41

System

**Temperature (°C):**	25,0	**Duration Used (s):**	60
**Count Rate (kcps):**	575,9	**Measurement Position (mm):**	3,00
**Cell Description:**	Disposable micro cuvette (4...	**Attenuator:**	9

Results

			**Size (d.nm):**	**% Volume:**	**St Dev (d.nm):**
**Z-Average (d.nm):**	22,75	**Peak 1:**	16,54	99,9	6,110
**PdI:**	0,254	**Peak 2:**	4998	0,1	881,0
**Intercept:**	0,817	**Peak 3:**	0,000	0,0	0,000
**Result quality**	Good				

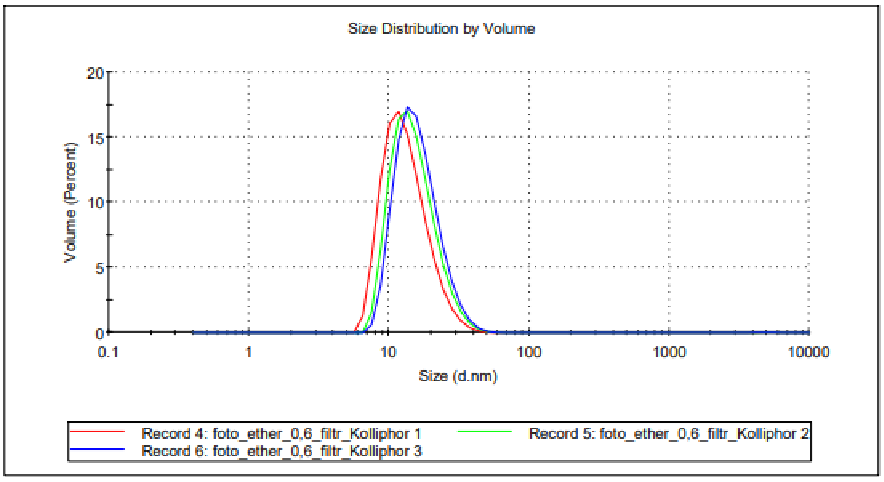

Figure S22. Size distribution report by volume for *E*-ethercaine (**6**) micellar solution.

Size Distribution Report by Volume

v2.2

Malvern

Sample Details

Sample Name: foto_ether_0_6_filtered_Kolliphor_UV

SOP Name: mansettings.nano

General Notes:

**File Name:**	151221.dts	**Dispersant Name:**	Water
**Record Number:**	36	**Dispersant RI:**	1,330
**Material RI:**	1,59	**Viscosity (cP):**	0,8872
**Material Absorbtion:**	0,010	**Measurement Date and Time:**	15 декабря 2021 г. 18:07:55

System

**Temperature (°C):**	25,0	**Duration Used (s):**	60
**Count Rate (kcps):**	417,0	**Measurement Position (mm):**	3,00
**Cell Description:**	Disposable micro cuvette (4...	**Attenuator:**	9

Results

			Size (d.nm):	% Volume:	St Dev (d.nm):
**Z-Average (d.nm):**	14,90	**Peak 1:**	10,45	100,0	4,074
**PdI:**	0,236	**Peak 2:**	4338	0,0	1224
**Intercept:**	0,823	**Peak 3:**	0,000	0,0	0,000
**Result quality**	Good				

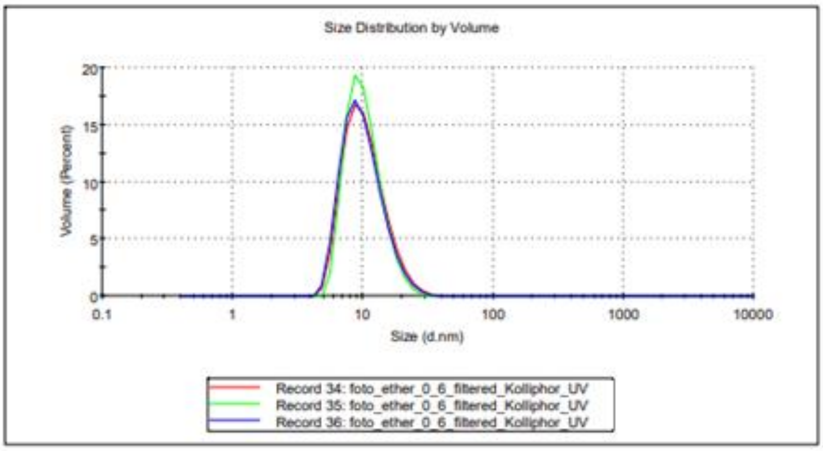

Figure S23. Size distribution report by volume for Z-ethercaine (**6**) micellar solution.
